# The path to the clinic: a comprehensive review on direct KRAS^G12C^ inhibitors

**DOI:** 10.1186/s13046-021-02225-w

**Published:** 2022-01-19

**Authors:** Albert K. Kwan, Gary A. Piazza, Adam B. Keeton, Caio A. Leite

**Affiliations:** 1 University of South Alabama College of Medicine, Mobile, AL USA; 2 Department of Drug Discovery and Development, Harrison School of Pharmacy, Auburn University, Auburn, AL USA; 3 Hospital BP, Beneficência Portuguesa de São Paulo, São Paulo, Brazil

**Keywords:** KRAS^G12C^, Cancer, Clinical trial, Direct RAS inhibitor, Sotorasib, Adagrasib, Epidemiology, Immunotherapy, Drug development

## Abstract

The *RAS* oncogene is both the most frequently mutated oncogene in human cancer and the first confirmed human oncogene to be discovered in 1982. After decades of research, in 2013, the Shokat lab achieved a seminal breakthrough by showing that the activated KRAS isozyme caused by the G12C mutation in the *KRAS* gene can be directly inhibited via a newly unearthed switch II pocket. Building upon this groundbreaking discovery, sotorasib (AMG510) obtained approval by the United States Food and Drug Administration in 2021 to become the first therapy to directly target the KRAS oncoprotein in any KRAS-mutant cancers, particularly those harboring the *KRAS*^G12C^ mutation. Adagrasib (MRTX849) and other direct KRAS^G12C^ inhibitors are currently being investigated in multiple clinical trials. In this review, we delve into the path leading to the development of this novel KRAS inhibitor, starting with the discovery, structure, and function of the RAS family of oncoproteins. We then examine the clinical relevance of KRAS, especially the KRAS^G12C^ mutation in human cancer, by providing an in-depth analysis of its cancer epidemiology. Finally, we review the preclinical evidence that supported the initial development of the direct KRAS^G12C^ inhibitors and summarize the ongoing clinical trials of all direct KRAS^G12C^ inhibitors.

## Background

Since the discovery of KRAS in the 1960’s, little progress has been made recently in treating patients with KRAS-driven cancers. After a period when directly targeting KRAS was considered unlikely, hence deeming KRAS “undruggable,” the Shokat lab reinvigorated the search for a direct KRAS inhibitor by discovering the KRAS^G12C^ switch II pocket in 2013. In May 2021, the United States Food and Drug Administration (FDA) approved sotorasib (Lumakras™) as the first treatment for adult patients with non-small cell lung cancer (NSCLC) harboring the *KRAS*^G12C^ genetic mutation who have received at least one prior systemic therapy. Thus, sotorasib became the first FDA-approved therapy to directly target the KRAS oncoprotein in tumors, particularly those with *KRAS*^G12C^ mutations. As of early August 2021, there have been twenty registered clinical trials investigating nine different direct KRAS^G12C^ inhibitors, with seven inhibitors currently being investigated.

The key purpose of this review is to convey a thorough account of the KRAS^G12C^ preclinical and clinical research landscape that has led to our present understanding of treating KRAS^G12C^-mutant cancers. We aim for this review to serve as a comprehensive primer for any physician or scientist interested in pursuing KRAS^G12C^ investigation. To our knowledge, we are not aware of any published review that has provided as extensive a coverage of all the landmark preclinical and clinical research regarding KRAS^G12C^ inhibition specifically.

## Discovery of *KRAS*

Discovered by its namesake in 1967, the Kirsten murine sarcoma virus (Ki-MSV) was first isolated as a sarcoma-inducing retrovirus during the passaging of murine leukemia viruses in rats [1]. In 1975, Scolnick et al. established Ki-MSV to be a recombinant virus with incorporated sequences derived from the rat genome [2]. Soon after, these sequences were molecularly characterized [3], and Ki-MSV was established to contain the rat cellular gene *Kras* [4].

Around the same time the retrovirus oncogene studies were conducted, DNA transfection studies were initially performed to unearth genes capable of inducing morphologic cellular transformation. In 1979, Weinberg et al. first reported the morphological transformation of NIH/3T3 mouse fibroblasts using genomic DNA isolated from chemically transformed rodent fibroblasts [5], which was followed by additional transformation studies with NIH/3T3 cells transfected with different human tumor cell lines by other groups [6–10].

In 1982, perhaps serendipitously, the Cooper, Weinberg, Barbacid, and Aaronson groups [11–13] implemented DNA hybridization with retroviral oncogene probes to determine that the human oncogenes which transformed the NIH/3T3 mouse fibroblast cell lines in the earlier transfection studies were homologous to the *RAS* genes identified in the Kirsten and Harvey sarcoma viruses. In fact, it was later revealed that the first NIH/3T3 transformants reported by the Weinberg group possessed an activated *KRAS* oncogene [14]. By the end of 1982, a single missense mutation in codon 12 was found to be the molecular basis of *HRAS* gene activation in the EJ/T24 bladder carcinoma cell line [15–17]. Moreover, the codon 12 mutation was found to be the activating mutation in *KRAS* in lung and colon tumor cells [18]. Mutant *RAS* genes were later identified in patient tumors but not in normal tissue, which validated the fact that the mutant *RAS* genes from aforementioned tumor cell lines were real and not artifacts of in vitro cell passage [19–21]. Thorough reviews describing the discovery of the *RAS* family have been previously published [19, 22, 23].

## Structure of RAS

Three genes (*HRAS*, *NRAS* and *KRAS*) encode the four major RAS isoforms: HRAS, NRAS, KRAS4A, and KRAS4B. *KRAS* encodes two variants due to alternative splicing of exon 4, which results in divergent C-terminal sequences. Both variants are expressed in human cells, but KRAS4B is the predominant isoform expressed in human cells while KRAS4A has low expression levels and is more similar to viral KRAS [19]. The amino-terminal residues 1–165 of the RAS proteins share 92–98% sequence identity. The remaining 23–24 carboxy-terminal residues diverge substantially in sequence and are thus defined as the hypervariable region (HVR) [24]. Although the protein sequences among RAS isomers are highly conserved, KRAS exhibits rare (i.e., genomically underrepresented) codon bias at the third (i.e., degenerate) position of codons: *KRAS* displays an A/T bias, resulting in relatively poor protein translation, while *HRAS* demonstrates an G/C bias. The degree of rare codon bias appears to be correlated with the mutation frequency among *RAS* isomers [25].

**Fig. 1:**
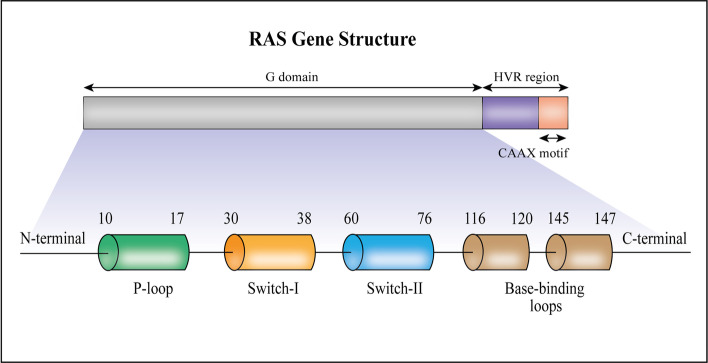
The G domain corresponds to the initial 166–168 residues. Bordering the nucleotide-binding pocket are four main regions: the phosphate-binding loop (P-loop, residues 10–17), switch I (residues 30–38), switch II (residues 60–76) and the base-binding loops (residues 116–120 and 145–147). The two switch regions regulate all known nucleotide-dependent interactions between RAS and its binding partners. The remaining residues in the carboxy-terminal constitutes the HVR, including the CAAX motif, a membrane anchor sequence. Abbreviations: HVR, hypervariable region; CAAX, C: cysteine amino acid, A: aliphatic amino acid, X: amino acid dictating whether farnesylated or geranylated

The initial 166–168 residues of the RAS proteins form a single-structured domain known as the G domain (Fig. 1), which is comprised of a mixed six-stranded β-sheet and five-α-helix fold that is typical for α,β-nucleotide-binding proteins [24]. Bordering the nucleotide-binding pocket are four main regions: the phosphate-binding loop (P-loop, residues 10–17), switch I (residues 30–38), switch II (residues 60–76) and the base-binding loops (residues 116–120 and 145–147) [24]. Described as a loaded-spring mechanism [26], the switch regions undergo conformational changes between the GDP- (guanosine diphosphate) and GTP-bound (guanosine triphosphate) states. In the GTP-bound state, threonine 35 (switch I region) and glycine 60 (switch II region) make hydrogen bonds with the γ-phosphate, holding the respective switch regions in their active conformations. Upon GTP hydrolysis and phosphate release, these two regions relax into their inactive GDP conformations. The two switch regions regulate all known nucleotide-dependent interactions between RAS and its binding partners [24].

The remaining residues in the carboxy-terminal constitute the HVR and lipid tail that appear to be poorly structured in solution. The HVR includes the CAAX (C: cysteine amino acid, A: aliphatic amino acid, X: amino acid dictating whether farnesylated or geranylated) motif, which is a membrane anchor sequence. Upon its initial synthesis in the cytosol, the RAS protein undergoes post-translational modifications to enable its association with the plasma membrane, which is essential for activation of its various downstream pathways. The subcellular localization of RAS proteins is determined by the specific lipid modification, composition of local membranes, and electrostatic nature of the isoform-specific HVR’s [27]. Once synthesized, RAS first undergoes prenylation, which involves farnesylation of the cysteine residue in the CAAX motif by fanesyltransferase. Only HRAS is exclusively prenylated by farnesyltransferase, while KRAS and NRAS (to a lesser extent) can undergo alternative prenylation by geranylgeranyl transferase-I when farnesyltransferase activity is blocked [28]. This alternative prenylation was discovered after the disappointing results of the promising clinical trials involving farnesyltransferase inhibitors (FTI’s) when targeting KRAS-driven tumors [29]. After farnesylation, the -AAX motif is cleaved by RAS-converting enzyme 1 (RCE1), and the farnesylated cysteine residue is then carboxymethylated by isoprenylcysteine carboxymethyltransferase (ICMT) [24].

All RAS proteins require a second signal for proper plasma membrane localization. Except for KRAS4B, this second signal includes palmitoylation at a second cysteine in the HVR, which creates a second hydrophobic anchor. Because KRAS4B lacks a second cysteine in the HVR, KRAS4B is not palmitoylated. Instead, the HVR of KRAS4B contains a polylysine region that anchors the protein to the plasma membrane via electrostatics. Similarly, KRAS4A contains a bipartite polybasic sequence that also contributes to membrane attachment via electrostatic interactions in addition to palmitoylation [24].

## Function of RAS

**Fig. 2:**
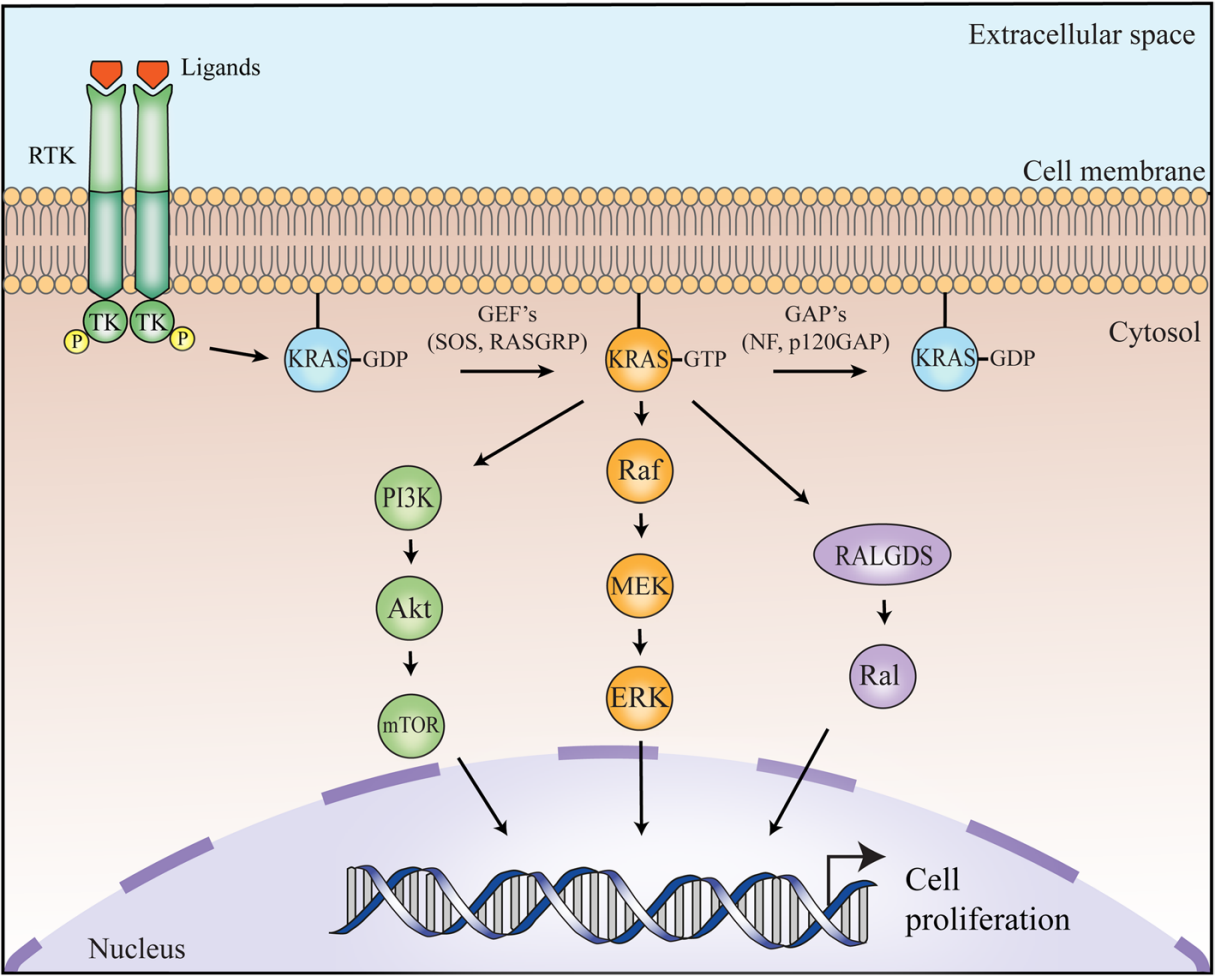
RAS proteins play a pivotal role in the regulation of cell proliferation, differentiation, and survival through various signal transduction cascades, including the canonical RAF–MEK–ERK/MAPK, PI3K–AKT–mTOR, and RALGDS–RAL pathways. Once the ligand binds to the extracellular domain of the RTK, the signal is transmitted through the transmembrane domain resulting in RTK dimerization and subsequent RAS activation. RAS signaling is further regulated by a balance between activation by GEF’s (e.g., SOS and RASGRP) and inactivation by GAP’s (e.g., NF and p120GAP). Abbreviations: RTK, receptor tyrosine kinase; GEF’s, guanine nucleotide exchange factors; GAP’s, GTPase-activating proteins; SOS, son of sevenless homologue; RASGRP, RAS guanyl nucelotide-releasing protein; NF, neurofibromin

RAS proteins are small, membrane-bound guanine nucleotide-binding GTPases. Functioning as binary switches, they cycle between their GTP-bound (active) and GDP-bound (inactive) conformations to regulate multiple signal transduction pathways. RAS proteins play a pivotal role in the regulation of cell proliferation, differentiation, and survival that drive multiple aspects of transformation and tumor progression through these signal transduction cascades, which include the canonical RAF–MEK–ERK/MAPK, PI3K–AKT–mTOR, and RALGDS–RAL pathways, among others (Fig. 2) [19, 30–35].

RAS signaling is regulated through a balance between activation by guanine nucleotide exchange factors (GEF’s) and inactivation by GTPase-activating proteins (GAP’s). RAS proteins intrinsically have a relatively slow off-rate for GDP (t_1/2_ = 6 min, k_off_ = 2 × 10^− 3^ s^− 1^ at 20°) [36]. Thus, GEF’s, such as son of sevenless homologue (SOS) and RAS guanyl nucelotide-releasing protein (RASGRP), are needed to accelerate the GDP/GTP exchange reaction by several orders of magnitude. Upon binding to GEF, RAS undergoes conformational changes in the switch regions and P loop that weaken the GDP affinity, ultimately resulting in GDP release and replacement by GTP [37]. Because RAS and GEF each have similar affinities for GDP or GTP, increased levels of GTP-bound RAS occur mainly due to the ~ 10-fold higher cellular GTP concentration of GTP relative to GDP. Binding of GTP dissociates the GEF and leads to the formation of an active GTP-bound RAS that can bind effectors. Moreover, RAS proteins have a relatively slow rate of intrinsic GTP hydrolysis (t_1/2_ = 16 min, k_off_ = 6 × 10^− 4^ s^− 1^) [36]. Therefore, for efficient GTP hydrolysis to occur, GAP’s, such as p120GAP and neurofibromin, function by accelerating the cleavage step by several orders of magnitude.

## Epidemiology of the RAS family in human cancer

### Overview of the RAS family

Numerous studies have reported mutant RAS as a key driver of transformation and tumor progression [19, 30, 38]. Cross-referencing data from the four leading cancer mutation databases Catalogue of Somatic Mutations in Cancer (COSMIC) [39], The Cancer Genome Atlas (TCGA) [40], the Memorial Sloan Kettering Cancer Center (MSKCC) cBioPortal [41], and the International Cancer Genome Consortium (ICGC) [42], as well as the private Foundation Medicine dataset, Prior and colleagues estimated ~ 19% of cancer patients to have a *RAS* mutation, which is equivalent to ~ 260,000 incident cancer cases in the United States alone [43]. Globally, there are ~ 18 million new cancer diagnoses per year [44], from which Prior and colleagues extrapolated to be ~ 3.4 million new cancer incidents with *RAS* mutations worldwide. Among mutations in the three *RAS* genes, gain-of-function missense mutations in *KRAS* account for the majority of *RAS* gene mutations (75%), followed by *NRAS* mutations (17%) and *HRAS* mutations (7%) [43, 45]. In pan-cancer analyses, mutations in any of these *RAS* family genes is associated with poor patient prognosis [41, 46].

The particular *RAS* gene mutated in cancer demonstrates a strong preference to its tissue of origin. In a given cancer type, *KRAS* mutations are the predominant *RAS* mutations found in pancreatic cancer (~ 88%). *KRAS* mutations are also notable in a variety of other cancer types including colon adenocarcinoma (50%), rectal adenocarcinoma (50%), lung adenocarcinoma (32%), small intestine adenocarcinoma (26%), cholangiocarcinoma (23%), plasma cell myeloma (18%), gallbladder carcinoma (16%), and anaplastic thyroid carcinoma (8.6%). Meanwhile, *NRAS* mutations are the most common *RAS* mutations in skin cutaneous melanoma (17%); hematologic malignancies including plasma cell myeloma (19%), acute myeloid leukemia (14%), chronic myeloid leukemia (9.7%), and acute lymphoblastic leukemia (9.6%); and endocrine thyroid malignancies including anaplastic thyroid carcinoma (19%), follicular thyroid carcinoma (19%), and papillary thyroid carcinoma (5.9%). *HRAS* mutations are mostly found in head and neck squamous cell carcinoma (5.1%) and bladder urothelial carcinoma (7%), and are also found in endocrine thyroid cancers to a lesser extent including follicular thyroid carcinoma (7%) [43].

*RAS* genes are further distinguished by their distinctive mutation frequency differences at three codon-specific hotspots, which also vary by tumor type. The most frequent sites of oncogenic mutation in RAS are residues G12, G13, and Q61. Residues G12 and G13 are located in the P-loop region of RAS, while residue Q61 is located in the switch II region. Single base missense mutations at these three key residues account for 99% of all *RAS* mutations [47]. Among KRAS mutations, G12 mutations are predominant (81%), followed by G13 mutations (14%) and Q61 mutations (2%) [43]. Among *NRAS* mutations, Q61 mutations are the majority (62%), followed by G12 mutations (23%) and G13 mutations (11%). HRAS mutations are more evenly split in residue preferences, but the preferences are still relatively apparent. Among HRAS mutations, Q61 mutations are the most prevalent (38%), followed by G12 mutations (26%) and G13 mutations (23%).

Furthermore, there are differences in the specific amino acid that is mutated at each of the three hotspots (G12, G13, and Q61), which also vary by tumor type (see below). Nineteen different activating mutations in codon 12, 13, or 61 can be created in each *RAS* isoform by a single base change. Among those three codons, five mutations (G12D, G12V, G12C, G13D and Q61R) account for 70% of all RAS-mutant cancers [43].

### Mutant KRAS in human cancer

**Fig. 3:**
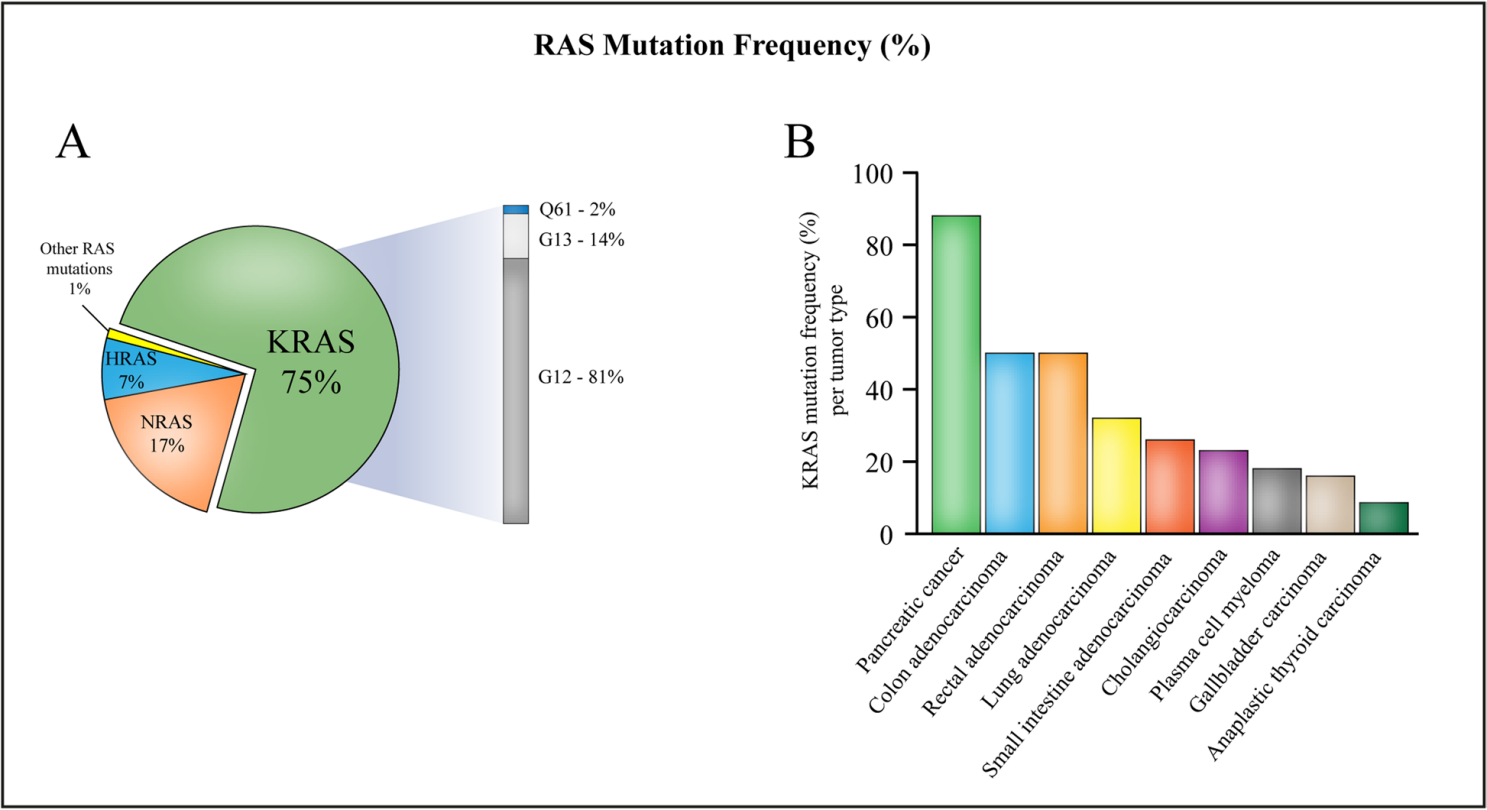
**A** The pie chart depicts the percentage of each *RAS* isoform contributing to all *RAS* mutations. Gain-of-function missense mutations in *KRAS* account for the majority of *RAS* gene mutations (75%), followed by *NRAS* mutations (17%) and *HRAS* mutations (7%). The remaining 1% represents RAS mutations that are other than gain-of-function missense mutations. Among *KRAS* mutations, the G12 codon (81%) is the most frequently mutated, followed by G13 (14%) and Q61 (2%). **B** The bar graph portrays the proportion of *RAS* mutations that are *KRAS* mutations found in a given cancer type. *KRAS* mutations are the most common *RAS* mutations in pancreatic cancer (~ 88%), followed by colon adenocarcinoma (50%), rectal adenocarcinoma (50%), lung adenocarcinoma (32%), small intestine adenocarcinoma (26%), cholangiocarcinoma (23%), plasma cell myeloma (18%), gallbladder carcinoma (16%), and anaplastic thyroid carcinoma (8.6%)

Globally, among the ~ 18 million new cancer diagnoses each year, ~ 2.6 million cancer patients are estimated to harbor a KRAS mutation (i.e., ~ 14% of all cancer cases) [43]. *KRAS* mutations alone account for ~ 1 million annual deaths worldwide, similarly to that for malaria and tuberculosis [28]. As stated earlier, gain-of-function missense mutations in *KRAS* account for the majority of *RAS* gene mutations in human cancer (75%). Among the 29 cancer types with *KRAS* mutations that were included in Table 1 of the study by Prior and colleagues, 43% of all *KRAS* mutations in human cancer are found in colorectal adenocarcinoma, followed by pancreatic adenocarcinoma (20%) and NSCLC adenocarcinoma subtype (14%). Within a particular cancer type, the three cancers in which *KRAS* mutations are the predominant *RAS* mutations are pancreatic cancer (~ 88%), colorectal adenocarcinoma (50%) [colon adenocarcinoma (50%) and rectal adenocarcinoma (50%)], and lung adenocarcinoma (32%). Because these three cancers alone are responsible for the majority of *KRAS* mutations found in human cancer (77%), much of the current focus on *KRAS*-mutant cancer therapeutics is targeted against these cancers. However, as illustrated in Fig. 3, *KRAS* mutations are also noteworthy in a variety of other cancer types including small intestine adenocarcinoma (26%), biliary tract cancers [cholangiocarcinoma (23%), gallbladder carcinoma (16%)], plasma cell myeloma (18%), and anaplastic thyroid carcinoma (8.6%).

Differences are observed in codon-specific and amino acid-specific mutation patterns that further vary depending on tumor type. Among all residue-specific KRAS mutations, the G12 mutation is the predominant mutation (81%), followed by the G13 mutation (14%) and Q61 mutation (2%) [43]. Among KRAS mutations in pancreatic adenocarcinoma, residue G12 mutations (94%) predominate, while G13 and Q61 mutations (1.2 and 2.4%, respectively) are rare [43]. On the other hand, among KRAS mutations in colon and rectal adenocarcinoma, G13 mutations are observed in relatively high frequencies (20 and 21%, respectively). Analyzing amino acid-specific KRAS mutations in the three cancer types responsible for most KRAS mutations, the most common KRAS mutation in NSCLC adenocarcinoma subtype is KRAS^G12C^ (glycine to cysteine) (33.4%), while the most common KRAS mutation in colon and rectal adenocarcinoma and pancreatic ductal adenocarcinoma is KRAS^G12D^ (glycine to aspartate) (32.7, 32.5, and 46.1%, respectively) [39].

Focusing on the KRAS^G12C^ mutant, this mutation is present in 12% of all KRAS-mutant cancers [39]. Prior et al. gathered data from COSMIC and American Cancer Society (ACS) 2018 cancer incidence statistics to estimate the number of KRAS^G12C^ mutations found in newly diagnosed cancer patients each year in the U.S. alone. They estimated that 32.8% of all KRAS^G12C^ mutations in human cancer are found most commonly in lung adenocarcinoma, followed by colorectal adenocarcinoma (18.8%) and pancreatic adenocarcinoma (4.6%) [43]. As a comparison, they estimated that KRAS^G12D^ mutant is most commonly found in pancreatic adenocarcinoma (29.9%), followed by colorectal adenocarcinoma (25.8%) and lung adenocarcinoma (5.9%) [43]. Analyzing the KRAS^G12C^ mutation frequencies in each of these three cancer types separately, Nassar et al. identified KRAS^G12C^ mutations in 13.8% of NSCLC (7.0% of lung adenocarcinoma-subtype [39]), 3.2% of colorectal cancer, and < 1% of pancreatic cancer patients using data extracted from the registry of the American Association for Cancer Research Project Genomics Evidence Neoplasia Information Exchange (GENIE), version 8.0 [48, 49]. In summary, these codon- and amino acid-specific mutation patterns that differ depending on the tumor tissue of origin must be taken into consideration when designing novel anticancer therapeutics since they may implicate distinct oncogenic properties and thus clinical consequences.

## Mechanistic and clinical implications of missense mutation-specific hotspots

Missense gene mutations at amino acid residues G12, G13, and Q61 have unique structural and functional consequences on the RAS protein, respectively [36, 38]. Substitution of glycine at G12 or G13 by any amino acid except proline are believed to cause a steric block that prevents the arginine finger of GAP from entering the GTPase site of RAS, thus discouraging hydrolysis as a result [50]. Because Q61 is part of the GTP hydrolysis mechanism, Q61 mutations abolish both intrinsic and GAP-mediated GTP hydrolysis. Overall, missense mutations that affect these residues result in increased RAS activation via GTP-loading, thus bypassing normal physiological activation by upstream input, for example, from receptor tyrosine kinases [28].

Mutagenesis studies first demonstrated the idea that certain amino acid substitutions at a given mutation hotspot can lead to varied functional consequences. In a study with HRAS as the model, a wide range of oncogenic outcomes was shown with all 19 possible amino acid substitutions at the G12 codon [51]. Additionally, in a similar study that examined the outcomes of 17 different mutations on HRAS Q61, all 17 mutants shared comparable defects in GTP hydrolysis activity in vitro, while the Q61P and Q61E mutations did not show increased transforming ability relative to HRAS WT [52]. In other studies, G12V mutation decreased GAP-mediated hydrolysis, Q61L mutation reduced both GAP-mediated and intrinsic hydrolysis and increased intrinsic nucleotide exchange, and G13D mutation reduced GAP-mediated hydrolysis and noticeably increased intrinsic nucleotide exchange [53]. In addition to specific mutations impacting direct RAS protein function, expanding evidence suggest mutation-specific consequences on the effector signaling pathways of RAS as well [54, 55].

To underscore the impact of the mutation-specific consequences on direct RAS protein function and its signaling pathways, Westover and colleagues profiled the biochemical and biophysical properties of commonly occurring KRAS mutants (G12A, G12C, G12D, G12R, G12V, G13D, Q61L, and Q61H) [36]. They analyzed the intrinsic and GAP-mediated GTP hydrolysis rates, GTP and GDP-binding kinetics, relative affinities for RAF kinase, and high-resolution crystal structures. Overall, mutations that affect the G12, G13 and Q61 residues resulted in decreased intrinsic and GAP-stimulated GTP-hydrolysis rates. Additionally, they showed that the G13D mutant expressed an increased rate of nucleotide exchange independent of GEF (e.g., SOS) regulation relative to the wild-type protein, which resulted in increased auto-activation and thus provided another potential mechanism of increased aberrant cellular RAS signaling.

Mutations impacting KRAS activity can be classified by having either a high (WT, G12C, G12D, G13D) or low (G12A, G12R, G12V, Q61L, and Q61H) level of intrinsic GTPase activity. Mutations can be further distinguished by having either high (WT, G12A, G12C, G13D, and Q61L) or low (G12R, G12V, and G12D) RAF affinity, based on their relative affinity for RAF kinase Ras-binding domain (RBD). With these criteria in mind, Westover and colleagues proposed a predictive model for the relative dependence on (i.e., activation of) the RAF kinase pathway compared with other canonical RAS signaling pathways such as PI3K and RalGDS among tumors with KRAS mutations. The Westover model predicts tumors with G12A and Q61L mutations preferentially signal through the RAF kinase pathway due to their high affinity for RAF kinase and relatively lower rates of intrinsic hydrolysis. In contrast, the model predicts G12D to have the lowest RAF activation levels due to its low affinity for RAF and faster hydrolysis rate. G12V and G12R would have moderate activation of RAF kinase due to their slow intrinsic hydrolysis rate coupled with a low RAF affinity. Likewise, G12C and G13D would also be predicted to have moderate activation of RAF kinase due to their high affinity but more rapid intrinsic GTPase activity that likely results in an attenuated duration of RAF kinase activation compared with G12A and Q61.

While the therapeutic response to farnesyltransferase inhibitors appears to be dependent on the specific RAS isoform mutated [29], there is increasing evidence that mutations at specific hotspots can also impact treatment outcomes among cancer patients. For example, based on clinical trials and various studies examining the efficacy of anti-epidermal growth factor receptor (EGFR) therapies such as cetuximab and panitumumab in treating colorectal cancer patients [56–61], the FDA revised their recommendation to exclude patients with KRAS G12 or G13 mutations from such treatment [62]. Currently, the National Comprehensive Cancer Network (NCCN) advises that colorectal cancer patients with any KRAS or NRAS mutation will not benefit from anti-EGFR therapy [63].

With increasing preclinical and clinical evidence supporting the importance of RAS isoform- and residue-specific mutation differences that are also both cellularly and genetically context-dependent [54], it appears for now that developing a single, universal anti-RAS therapeutic approach that specifically targets RAS mutants, without affecting RAS wild-type, in cancer is a challenging task. Thus, a pan-KRAS [64] or pan-RAS [65] inhibitor may have distinct advantages. For the foreseeable future, a targeted therapeutic approach that includes combinatory strategies and is dependent on both the RAS mutation subset and its environmental context seems to be the next step in optimally treating patients with RAS-driven cancers.

## The path to the clinic: how it began

### Introduction

Prior to the seminal discovery by Shokat and colleagues in 2013, KRAS was considered undruggable for several reasons. Firstly, the molecular structure of KRAS has intrinsically shown high resistance to small molecule modulation. Small molecule inhibitors, by design, primarily bind to identifiable pockets within a protein. However, KRAS is a small protein with a relatively smooth surface. Except for its GTP/GDP-binding pocket, the KRAS protein does not appear to have any well-defined, hydrophobic pockets viable for small molecule inhibitor binding. Furthermore, under the physiological conditions, GTP almost exclusively occupies GTP/GDP-binding pocket with extremely high affinity in the picomolar range. Coupled with the relatively high cellular GTP concentrations in the micromolar range (~ 500 μM), designing a small molecule inhibitor that can achieve adequate intracellular concentration to compete with GTP at the GTP/GDP-binding pocket appeared formidable. Examining the successful advent of tyrosine kinase inhibitors, as a comparison, the binding affinity with their natural co-substrate ATP is in the micromolar range (a million-fold difference in binding affinity), and ATP cellular concentrations range from 1 to 10 mM. Taking these factors into consideration, direct targeting of KRAS by small molecule inhibitors has been a challenging task [24, 31, 34, 47, 66–70].

Because of these challenges, therapeutic approaches primarily focused on targeting KRAS indirectly. These indirect therapeutic strategies include targeting downstream or upstream RAS signaling pathways, metabolic pathways, and synthetic lethality approaches, all of which have yielded relatively disappointing results. Time and time again, indirect inhibition strategies have resulted in disappointing clinical trial outcomes in treating KRAS-mutant cancers [30, 31, 47, 66–68, 71–80]. Efficacy with these treatments were lacking due to subsequent unexpected resistance mechanisms, as observed with the farnesyltransferase, BRAF, and EGFR inhibitors. However, in 2013, the groundbreaking foundation set by Shokat and colleagues renewed hope for direct KRAS inhibition, paving way for pharmacotherapeutics such as AMG510 and MRTX849 now currently being investigated in clinical trials with hopeful preliminary outcomes.

### Discovery of the KRAS^G12C^ inhibitor and the switch II pocket (S-IIP)

**Fig. 4:**
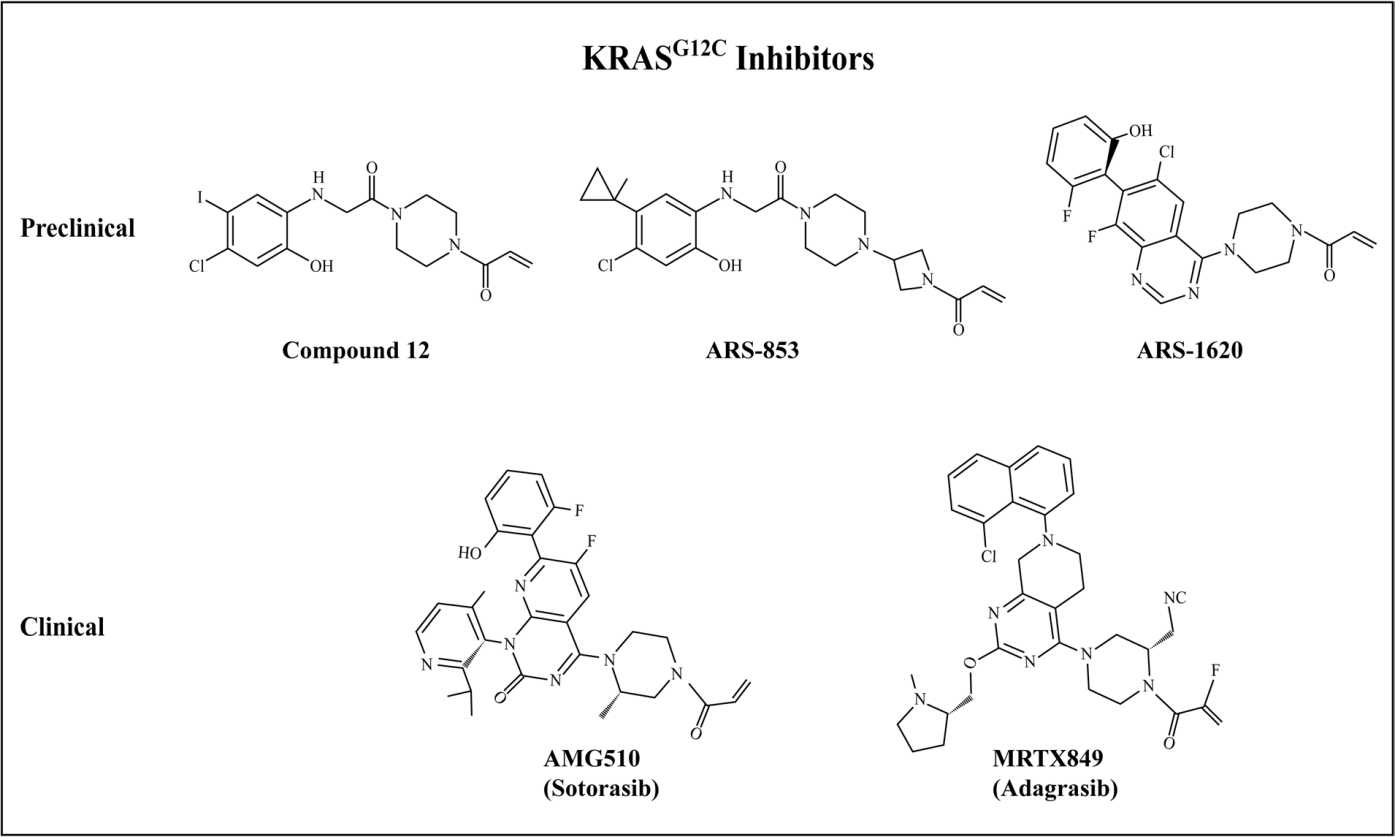
This figure illustrates the chemical structures of the first direct KRAS^G12C^ inhibitors in the preclinical and clinical years. Compound 12 was the initial lead compound developed by the Shokat lab; it was subsequently optimized to ARS-853, the first direct small molecular inhibitor shown to selectively inhibit KRAS^G12C^ in cells with potency in the range of a drug candidate. Introduction of a quinazoline core and a fluorophenol hydrophobic binding moiety resulted in ARS-1620, the first drug candidate to demonstrate in vivo potency. In May 2021, AMG510 (sotorasib) became the first FDA-approved therapy to directly target *KRAS*-mutated tumors. In June 2021, MRTX849 (adagrasib) received breakthrough therapy designation by the FDA, driving MRTX849 nearer to entering the clinic as well

In 2013, Ostrem, Shokat, and colleagues made a breakthrough by successfully developing a series of compounds that covalently and irreversibly bound to the cysteine residue of the KRAS^G12C^ (glycine to cysteine substitution) mutant, the most potent compound being compound 12 (Fig. 4) [24]. Creatively, they used a disulfide tethering approach that enabled identification of small molecular fragments that could noncovalently bind to shallow pockets with low affinity under reducing conditions and covalently tether with cysteine residues. With this approach, they identified compounds that bound to a newly discovered allosteric pocket beneath the switch II region near the mutant cysteine, which they named the switch II pocket (S-IIP). This highly dynamic allosteric pocket was not apparent in the apo-crystal structures but appeared to be induced or stabilized by the compound. These compounds preferentially bound to RAS in the GDP-bound state, decreased RAS affinity for GTP relative to GDP, impaired SOS-catalyzed nucleotide exchange from GDP to GTP, and blocked RAS-RAF effector interaction in the KRAS^G12C^ mutant cells. The blockade of RAS–RAF effector interaction may be attributed to a combination of inhibitor effects on nucleotide exchange, relative nucleotide affinities, and perhaps most critically, conformation effects on switch I and switch II. Importantly, by depending on the mutant cysteine residue for binding, the compounds specifically target the KRAS^G12C^ mutant protein while sparing KRAS wild-type protein, an observation supported by the selectively inhibition of growth of cancer cell lines expressing KRAS^G12C^. Specifically targeting KRAS mutants over KRAS wild-type would, in theory, minimize the toxicities and side effects associated with a potential pharmacotherapeutic agent, thus greatly enhancing its therapeutic index when treating cancer patients.

Examining the mechanism of action of the mutant-specific inhibitors in detail, by covalently binding to mutant cysteine in the S-IIP, the compounds were found to displace glycine 60, located in the switch II region (residues 60–76), towards the switch I region (residues 30–38). The displacement resulted in the disordering of the switch I region and loss of Mg^2+^ in several co-crystal structures produced by the Shokat lab. The GTP-bound state of RAS is quite sensitive to conformational disturbances in the regions of glycine 60 (switch II region) and threonine 35 (switch I region), demonstrated by the dominant-negative effects of the conservative mutants RAS^G60A^ and RAS^T35S^. A co-crystal structure in the GTP state was unable to be obtained to confirm the conformational changes of the switch I and switch II regions upon inhibitor binding, but the presence of the inhibitor in the S-IIP would have pronounced effects on the positions of these two described residues [24].

Preferential binding to GDP-bound RAS may appear to be an issue because it contradicts the widely held perception that RAS mutants in cancer are deficient in both intrinsic and GAP-stimulated GTP hydrolysis activity, resulting in RAS mutants to be in the constitutively active, GTP-bound state independent of GEF activity at steady state. After all, codon 12 mutations are known to disable the activation of intrinsic RAS GTPase activity by GAP’s. However, this perception has been gradually dispersed as new evidence emerged in recent years. KRAS^G12^ mutants have relatively high intrinsic GTPase activity (t_1/2_ = ~ 23–27 min) that is similar to that of wild-type RAS proteins [36, 81, 82]. Biochemical and biophysical analyses by Westover and colleagues have shown that different RAS mutants exhibit variable rates of intrinsic and GAP-stimulated GTP hydrolysis, as well as variable intrinsic and GEF-mediated nucleotide exchange [36]. These discrepancies result in concentration differences in GTP-bound KRAS between KRAS mutations, even at a specific residue. Moreover, additional studies have strengthened the possibility that the nucleotide-bound state of RAS is in constant flux, instead of a single, constitutively active state (see below) [82, 83]. These data may explain why the compounds are able to specifically bind to the inactive, GDP-bound KRAS mutants to ultimately reduce the level of the active, GTP-bound KRAS mutants in the cell.

## The preclinical years

### ARS-853

Despite its initial success, the initial lead compound (compound 12) developed by the Shokat lab had suboptimal pharmacologic properties, with indeterminate potency against the KRAS^G12C^ mutant in a cellular context. Indeed, two subsequent studies by Patricelli et al. and Lito et al. demonstrated that compound 12 was not capable of engaging KRAS^G12C^ in cells even at a relatively high dose and long incubation time [82, 83]. Compound 12 was eventually licensed to Wellspring Biosciences, a subsidiary of Araxes Pharma (both co-founded by Kevan Shokat in 2012). Further optimization of this scaffold resulted in an improved inhibitor, ARS-853, the first direct KRAS inhibitor shown to selectively inhibit KRAS in cells with potency in the range of a drug candidate [82].

ARS-853 had a very similar chemical structure to compound 12, which further supported the potential to inhibit the S-IIP [24], and also only covalently bound KRAS^G12C^ in the GDP-bound state. Target engagement by ARS-853 was demonstrated to be both time- and concentration-dependent, as expected for irreversible covalent interactions. In comparison to compound 12, the newly improved inhibitor rapidly bound KRAS^G12C^, increasing the reaction rate in a biochemical assay by 600-fold; bound KRAS^G12C^ at lower micromolar concentrations in both a biochemical and cellular context; and blocked nucleotide exchange (GDP to GTP) with much higher efficacy. ARS-853 potently inhibited the growth of KRAS^G12C^ mutant cells, but not non-KRAS^G12C^ cells, in ultra-low adherent 3D culture. Using mass spectrometry, Patricelli et al. and Lito et al. demonstrated specific binding of ARS-853 to KRAS^G12C^ in cells and showed subsequent blockade of downstream signaling through the RAF–MEK–ERK and PI3K–AKT pathways, which is most likely due to failure of GTP loading onto RAS. The authors found no effects of ARS-853 on RAS signaling or growth in non-KRAS^G12C^ cells at concentrations up to 10-fold higher than its KRAS^G12C^ potency. Moreover, across the 2740 surface-exposed cysteine residues profiled from 1584 proteins, KRAS^G12C^ was the most potent target of ARS-853, with only two off-targets (FAM213A and Reticulon-4) that also showed significant engagement with ARS-853. In summary, the studies established ARS-853 to be a selective, covalent inhibitor with low micromolar potency in cancer cells harboring G12C mutations in KRAS [82, 83].

As mentioned earlier, there is increasing evidence that KRAS mutants are not in a constitutively active, GTP-bound configuration at steady state. Rather, RAS mutants exhibit variances in intrinsic nucleotide exchange and GTPase activity, and GEF- and GAP-mediated cycling, that ultimately create concentration differences of GTP-bound (and GDP-bound) RAS within a cell. These variances depend on the RAS codon mutation, even down to the specific residue that is mutated. The studies on ARS-853 and direct KRAS^G12C^ inhibition conducted by Patricelli et al. and Lito et al. provide further support for the plasticity of the nucleotide cycle of KRAS mutants. When second-site substitution mutations were introduced that accelerated intrinsic nucleotide exchange, a decrease in ARS-853-mediated KRAS^G12C^ inhibition was observed. Furthermore, when second-site substitution mutations were introduced that decreased the intrinsic rate of nucleotide hydrolysis, ARS-853-mediated inhibition of KRAS^G12C^ was prevented.

Observing that the KRAS mutant had intrinsic GTPase hydrolysis and nucleotide exchange activity, Patricelli et al. and Lito et al. assessed if increased cellular GEF activity regulated by upstream receptor tyrosine kinases (RTK’s) attenuated ARS-853 engagement with KRAS^G12C^. Treatment with the EGFR inhibitor erlotinib increased ARS-853 engagement with KRAS, which one would expect since treatment would result in an increased GDP/GTP-bound ratio of KRAS mutants due to the decrease in GEF activity, given that KRAS mutants truly do not exist in a constitutively active GTP-bound state. Furthermore, the impact of inhibiting downstream effectors of the RAS signaling pathway on ARS-853-mediated KRAS^G12C^ inhibition was investigated. Treatment with the MEK inhibitor trametinib increased GTP-bound KRAS^G12C^ levels and decreased the efficacy of ARS-853 in cells, possibly through the release of ERK-mediated negative feedback inhibition of the SOS1 GEF. Taken together, these findings further strengthen the possibility that KRAS^G12C^ retains appreciable intrinsic GTPase activity and nucleotide exchange such that it is not fully GTP bound in vivo. Moreover, the studies revealed a potential mechanism of acquired resistance against direct KRAS^G12C^ inhibitors via increased upstream signaling by RTK-mediated GEF activity, hence supporting combination therapy with direct or indirect GEF inhibitors, such as EGFR inhibitors, in KRAS^G12C^ mutant cancers.

### ARS-1620

Although ARS-853 was the first KRAS^G12C^ inhibitor with cellular potency in the range of a drug candidate, it lacked adequate potency in vivo in mouse tumor models. A major drawback of ARS-853 was the short metabolic plasma stability and poor bioavailability. Thus, the inherent poor chemical and metabolic stability of ARS-853 series made it unsuitable for further preclinical development.

Subsequently, Janes et al. reported a new generation of KRAS^G12C^ S-IIP inhibitors, ARS-1620 [84]. ARS-1620 was a direct KRAS^G12C^ small molecule inhibitor that is potent, selective, orally bioavailable, and well-tolerated in mice. The compound exhibited both in vitro and in vivo potency with a therapeutic window in the range of a drug candidate. With improved potency and pharmacologic properties, ARS-1620 was highly efficacious as a single agent in multiple human cancer cell line- and patient-derived mouse xenograft tumor models. Thus, ARS-1620 provided the first in vivo evidence that the S-IIP targeted approach may be a promising therapeutic strategy for patients with *KRAS p.G12C* mutant cancers.

As an interesting aside, Janes et al. noted that in vitro studies assessing KRAS dependency using monolayer (2D-adherent) cell cultures significantly underestimated KRAS dependence in vivo*,* since they found that 3D ultra-low adherent suspension spheroid cultures better predicted in vivo sensitivity of KRAS mutant cancer cells to ARS-1620. The authors were not aware of any approved oncology drugs that displayed differential activity between 2D and 3D cultures as substantial as KRAS inhibition. These findings have significant translational implications for interpreting in vitro synthetic lethal relationships of *KRAS* as a driving oncogene. However, the value of using 3D cultures to predict clinical response to KRAS^G12C^-directed inhibitors needs to be validated by patient response rates in clinical trials.

### AMG510

Prior to the Shokat lab’s initial disclosure of their efforts targeting KRAS^G12C^, Amgen had also independently initiated their own research program to identify covalent inhibitors of KRAS^G12C^ [85]. In collaboration with Carmot Therapeutics, Amgen utilized Carmot’s Chemotype Evolution platform, a custom library synthesis and screening platform, and structure-based design to identify a series of selective covalent inhibitors of KRAS^G12C^, which ultimately resulted in advanced lead 1 that potently inactivated KRAS^G12C^ in biochemical and cellular assays [86]. Similar to ARS-1620, advanced lead 1 also attached to the S-IIP and covalently bound to mutant cysteine. However, cocrystallization of advanced lead 1 with GDP-bound KRAS^G12C^ revealed advanced lead 1 to occupy a previously unexploited cryptic pocket on the surface of KRAS [85], which was first independently identified earlier by Gentile et al. [87]. The hidden pocket is bordered by the H95, Y96, and Q99 side chains, and was unveiled by rotation of the H95 (histidine 95) side chain. Engagement of this cryptic pocket led to a multifold enhancement in cellular potency relative to ARS-1620. However, advanced lead 1 was not suitable for preclinical development due to its high clearance and low oral bioavailability in vivo in rodent model systems. Subsequently, superposition of binding modalities of advanced lead 1 and ARS-1620 led to the substitution of the quinazoline nitrogen (N1) of ARS-1620 as an alternative approach to exploit the H95/Y96/Q99 cryptic pocket. After further improvements, compound (R)-38 was created with enhanced potency and optimized pharmacokinetics suitable for preclinical development, and it was later nominated as a clinical candidate and coded as AMG510.

In a series of experiments, Canon et al. demonstrated AMG510 to be potent, efficacious, and selective in both cellular and mouse tumor models [88]. Treatment resulted in regression of KRAS^G12C^-specific tumors and decreased downstream p-ERK signaling. Sharing structural similarities with ARS-1620, AMG510 selectively bound to KRAS^G12C^, not KRAS wild-type, in the S-IIP region and covalently bound to mutant cysteine as well. However, unlike ARS-1620, AMG510 additionally bound to the His95 groove. One prominent liability of ARS-1620 is its suboptimal potency resulting from the small volume of the S-IIP that it occupies, which provided limited avenues for additional protein–ligand interactions. In contrast, the isopropyl-methylpyridine substituent of AMG510 occupying the His95 groove engaged in a continuous network of 25 ligand–protein van der Waals contacts, which extends from the backbone of helix 2 (His95, Tyr96) to the backbone of the flexible switch II loop. These interactions enhanced the potency of AMG510 by approximately 10-fold [mean half-maximum inhibitory concentration (IC_50_) = 0.09 μM)] compared to ARS-1620 in a nucleotide-exchange assay with recombinant GDP-bound KRAS^G12C^.

Additionally, Canon et al. examined various combination therapies with AMG510 to assess if combination therapy could overcome potential resistance and enhance tumor-cell killing. Referencing the clinically validated strategy of combining BRAF and MEK inhibitors in treating melanoma [89], they investigated combinations of AMG510 and various inhibitors of the MAPK and AKT signaling pathways, including inhibitors of HER kinases, EGFR, SHP2, PI3K, AKT and MEK. Multiple combinations resulted in synergistic activity, especially with the MEK inhibitor trametinib, a combination that demonstrated significantly enhanced anti-tumor activity in monolayer and spheroid cell culture models as well as in mouse tumor models compared to using either agent alone. Given the prevalence of KRAS^G12C^ mutations in lung adenocarcinoma, the authors also investigated combination treatment of AMG510 with carboplatin, a standard-of-care chemotherapeutic for lung cancers. While both drugs alone significantly inhibited tumor growth in KRAS^G12C^-mutant NCI-H358 (NSCLC) xenograft mice models, combination therapy resulted in greater anti-tumor activity. In summary, these preclinical data provide support for the clinical combination of AMG510 with MAPK inhibitors or chemotherapeutic agents to potentially bypass compensatory mechanisms of resistance, increase potency of therapeutic effects, and create a durable response to treatment.

Moreover, the authors investigated the role of the immune system in affecting the efficacy of AMG510 in treating KRAS^G12C^-mutant cancers. They reported that AMG510 induced long-term cures in immunocompetent mice with KRAS^G12C^-mutant CT-26 (colorectal) xenograft mice models, and induced tumor regression but not cures in the same mice tumor models lacking T cells (immunocompromised). Thus, they examined a combination strategy with immunotherapy, specifically with anti-PD-1 immune checkpoint inhibition since immune checkpoint blockade involving programmed cell death protein 1 (PD-1) and programmed death-ligand 1 (PD-L1) has been clinically validated in multiple settings [90, 91]. As monotherapy, AMG510 or anti-PD-1 inhibition caused complete tumor regression in only one of ten mice, respectively. However, combination treatment resulted in significantly improved survival, with complete and durable tumor regression in nine out of ten mice.

Analyzing the treatment effects on the immune cell composition of the tumor microenvironment, Canon et al. reported that AMG510, alone and in combination with anti-PD-1 inhibition, promoted a pro-inflammatory tumor microenvironment. Notably, there was increased T cell infiltration, primarily cytotoxic CD8^+^ T cells, into the tumor in both treatment arms relative to anti-PD-1 inhibition alone. Additionally, AMG510 treatment led to increased number of total and proliferating CD3^+^ T cells and total CD8^+^ T cells, further increased with combination therapy; and increased infiltration of innate immune system macrophages and dendritic cells, including CD103^+^ cross-presenting dendritic cells, which are critical in T cell priming and activation and are also implicated in T cell recruitment [92]. As an additional comparison, AMG510 was also compared with a MEK inhibitor. Both inhibitors regressed CT-26 KRAS^G12C^ tumors in mice to similar level, but unlike AMG510, MEK inhibition did not significantly affect the numbers of infiltrating CD8^+^ T cells.

To study a potential mechanism of action of immune system upregulation in the tumor microenvironment, transcriptional profiling of tumor RNA post-treatment with AMG510 revealed increased interferon signaling, chemokine production, antigen processing, cytotoxic and natural killer cell activity, and markers of innate immune system stimulation. Notably, there was increased expression of chemokines *Cxcl10* and *Cxcl11*, two key attractants of tumor-suppressive immune cells, that may explain the enhanced immunosurveillance after AMG510 treatment [92, 93].

Finally, in contrast to previous data suggesting that non-tumor-selective MEK inhibition in combination with anti-PD-1 treatment blocked T cell expansion and priming in vivo despite anti-tumor activity [94], Canon et al. found that AMG510 increased T cell priming, antigen recognition of tumor cells, and led to potential establishment of long-term tumor-specific T cell adaptive immune responses. The authors showed that AMG510 increased the expression of MHC class I antigens on tumor cells in vitro. Furthermore, combination therapy of AMG510 and anti-PD-1 established a memory T cell response against tumor cells in vivo. In the study, CT-26 KRAS^G12C^ mice cured by combination treatment of AMG510 and anti-PD-1 were rechallenged with CT-26 KRAS^G12C^, parental CT-26 (KRAS^G12D^), or 4 T1 (unrelated breast tumor model) tumor cells. None of the KRAS-mutant cell lines grew tumors, while tumors formed in all non-KRAS-mutant (4 T1) cells. There was a ~ three-fold increase in IFNγ secretion, a marker of tumor-specific T cell priming and activity, when splenocytes collected from the cured mice were stimulated by the two KRAS-mutant tumor cell lines, but no IFNγ induction was observed in the non-KRAS-mutant cell line. Together, these data suggest that AMG510 and anti-PD-1 combination therapy can establish an adaptive immune response against shared antigens that can recognize and eliminate related but non-KRAS^G12C^ tumors as well, a clinically relevant outcome since intratumoral KRAS mutation status can be heterogeneous within the same tumor and between primary and metastatic sites [95–100].

### MRTX849

In collaboration with Array BioPharma, Mirati Therapeutics conducted a covalent fragment screen of the Array BioPharma collection and identified a series of tetrahydropyridopyrimidines as irreversible covalent inhibitors of KRAS^G12C^, which led to the discovery of compound 4 [101]. Despite originating from an independent fragment screen, compound 4 markedly resembled the previously reported KRAS^G12C^ acrylamide inhibitors ARS-1620 and AMG510 [102]. With structure-based design, compound 4 was further optimized to compound 13 [101]. While compound 13 potently inhibited KRAS^G12C^ in animal models and led to tumor regression via intraperitoneal dosage, it suffered from rapid clearance and very low oral bioavailability intravenously. Subsequent tests identified the naphthol and acrylamide groups as the most metabolically sensitive positions in the molecule. Consequently, modifications of the groups were made that ultimately gave rise to MRTX849, which displayed much better stability and bioavailability [103].

Hallin et al. provided detailed characterization of MRTX849 activity, elucidation of response and resistance mechanisms, and identification of effective combinations [104]. Conducting cell viability assays using *KRAS*^*G12C*^-mutant cell lines in vitro, they found IC_50_ values ranging between 10 and 973 nmol/L in the 2-D monolayer cultures and between 0.2 and 1042 nmol/L in the 3-D spheroid cultures, suggesting a differential degree of sensitivity to treatment they found to be at least partially attributed to KRAS-dependent ERK and S6 signaling. In vivo, in a panel of human *KRAS*^*G12C*^ mutant cell line–derived xenograft and patient-derived xenograft models, MRTX849 demonstrated broadly active tumor regression exceeding 30% volume reduction from baseline in 17 of 26 models (65%). In addition, the authors found that baseline RNA sequencing, reverse phase protein array analysis, nor co-occurring alterations were sufficient to predict activity or response variability to therapeutic intervention. To examine mechanisms of therapeutic response and resistance and explore possible synergistic vulnerabilities for combination therapy, the authors performed CRISPR/Cas9, targeting 400 genes, and pharmacologic screening on several *KRAS*^*G12C*^ models. Both screens resulted in hits converging on regulators of the RAS pathway [(e.g., *SHP2/PTPN11*, receptor tyrosine kinases (RTK)], mTOR pathway, and cell cycle. These data were consistent with the more limited prior studies with the ARS-1620 KRAS^G12C^ inhibitor [105, 106]. In a subsequent series of in vivo experiments, combination therapy with MRTX849 was examined with afatinib (pan-HER inhibitor), palbociclib (CDK4/6 inhibitor), vistusertib (ATP-competitive mTOR inhibitor), and RMC-4550 (SHP2 inhibitor). Overall, results showed augmented responses that led to substantial tumor regression in multiple tumor models, including those that were refractory to MRTX849 single therapy.

## The clinical years

### JNJ-74699157 (ARS-3248)

**Fig. 5:**
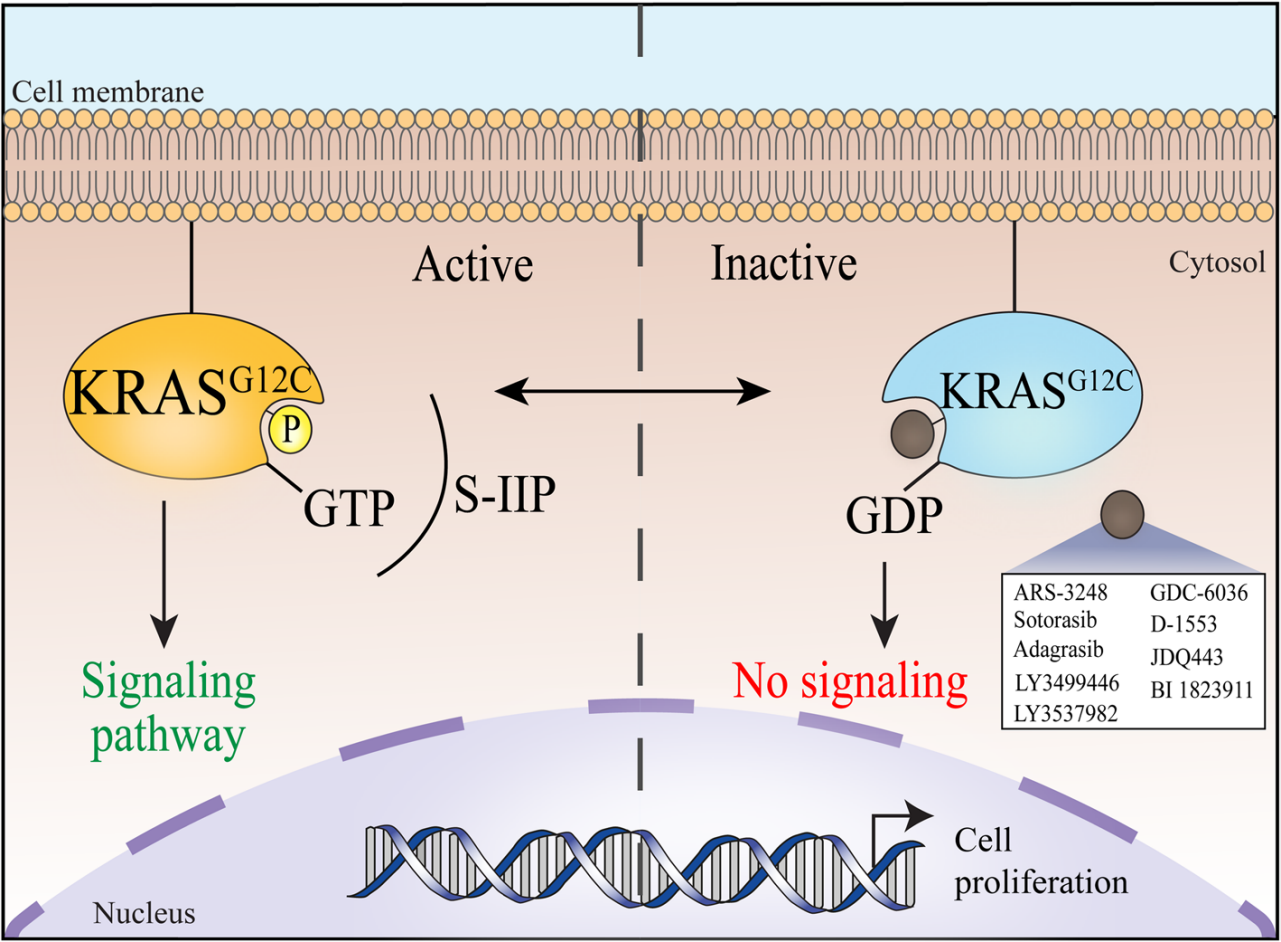
The diagram illustrates the nine small molecular inhibitors in registered clinical trials that directly target the KRAS^G12C^ mutant by binding to the switch II pocket (S-IIP). These inhibitors preferentially bind and stabilize RAS in the GDP-bound state, ultimately resulting in decreased signal transduction, especially by the RAF-MEK-ERK/MAP pathway, and thus preventing tumor progression

In February 2013, Wellspring Biosciences, a subsidiary of Araxes Pharma (both co-founded by Shokat and colleagues), entered a drug discovery and development agreement with Johnson & Johnson’s Janssen Biotech to further develop direct KRAS^G12C^ inhibitors for the treatment of cancer. Their collaboration led to the creation of ARS-3248, a new generation of KRAS^G12C^ inhibitor building upon ARS-1620. ARS-3248 is an investigational, orally available small molecule designed to potently and selectively inhibit KRAS^G12C^. JNJ-74699157 (ARS-3248) entered clinical trials in July 2019 (ClinicalTrials.gov number NCT04006301, Table 1), and the trial was completed in November 2020, with no results currently posted (Fig. 5).

**Table 1:** Direct KRAS^G12C^ Inhibitors in Clinical Trials

Agent	Company	Identifier	Phase	Status	Details
JNJ-74699157 (ARS-3248)	Janssen Research & Development, LLC	NCT04006301	1	Completed	Non-randomized, open-label study examining monotherapy in patients with advanced solid tumors (e.g., NSCLC, CRC) harboring the KRAS^G12C^ mutation. Results unavailable.
AMG510 (sotorasib)	Amgen	NCT03600883	1/2	Recruiting	CodeBreaK100. Randomized, open-label study examining AMG510 alone and in combination with anti (PD-1/L1) in patients with advanced solid tumors with KRAS p.G12C mutation, and AMG510 alone in patients with naïve (i.e., previously untreated) advanced NSCLC with KRAS p.G12C mutation.
		NCT04185883	1b/2	Recruiting	CodeBreaK101. Non-randomized, open-label study assessing AMG510 alone and in combination with other anti-cancer therapies in patients with advanced solid tumors with KRAS p.G12C mutation. Treatment arms include monotherapy in patients with advanced NSCLC with brain metastases; in combination with trametinib (MEK1/2 inhibitor), AMG 404 (PD-1 inhibitor), RMC-4630 (SHP2 inhibitor), palbociclib (CDK4/6 inhibitor), or everolimus (mTOR inhibitor) in patients with advanced solid tumors; in combination with afatinib (pan-ErbB inhibitor), pembrolizumab (PD-1 inhibitor), AMG 404 (PD-1 inhibitor), atezolizumab (PD-L1 inhibitor), or chemotherapy (carboplatin, pemetraxed, and docetaxel) in patients with advanced NSCLC; in combination with panitumumab (EGFR inhibitor) +/− chemotherapy (FOLFIRI), trametinib and panitumumab, or bevacizumab-awwb and chemotherapy (FOLFIRI or FOLFOX) in patients with advanced CRC.
		NCT04380753	1	Recruiting	CodeBreaK105. Open-label study assessing monotherapy in patients of Chinese ancestry with advanced/metastatic solid tumors with KRAS p.G12C mutation.
		NCT04303780	3	Active, not recruiting	CodeBreaK200. Randomized, open-label study comparing monotherapy vs. docetaxel in patients with previously treated advanced/metastatic NSCLC with KRAS p.G12C mutation.
		NCT04933695	2	Not yet recruiting	CodeBreaK201. Randomized, open-label study investigating monotherapy in patients with Stage IV NSCLC with KRAS^G12C^ mutation and PD-L1 TPS < 1%, stratified by STK11 co-mutation.
		NCT04625647	2	Not yet recruiting	Lung-MAP Treatment Trial sub-study (S1900E). Open-label study evaluating monotherapy in patients with stage IV or recurrent non-squamous NSCLC with KRAS^G12C^ mutation.
		NCT04667234		Expanded access (Available)	Study provides expanded access to and characterize the safety profile of AMG 510 in patients with previously treated locally advanced/unresectable/metastatic NSCLC with KRAS p.G12C mutation in a real-world setting.
MRTX849 (adagrasib)	Mirati Therapeutics Inc.	NCT03785249	1/2	Recruiting	KRYSTAL-1. Non-randomized, open-label clinical trial evaluating MRTX849 alone in patients with advanced/metastatic solid tumors with KRAS^G12C^ mutation, in combination with pembrolizumab or afatinib in patients with NSCLC with KRAS^G12C^ mutation, or in combination with cetuximab in patients with CRC with KRAS^G12C^ mutation.
		NCT04330664	1/2	Recruiting	KRYSTAL 2. Non-randomized, open-label study investigating MRTX849 in combination with TNO155 (SHP2 inhibitor) in patients with advanced solid tumors (NSCLC or CRC) with KRAS^G12C^ mutation.
		NCT04613596	2	Recruiting	KRYSTAL-7. Open-label study examining MRTX849 in combination with pembrolizumab (anti-PD1 antibody) in patients with advanced NSCLC with KRAS^G12C^ mutation.
		NCT04793958	3	Recruiting	KRYSTAL-10. Randomized, open-label study comparing combination therapy MRTX849 and cetuximab (anti-EGFR antibody) vs. chemotherapy (mFOLFOX6 or FOLFIRI) in patients with advanced/metastatic CRC with KRAS^G12C^ mutation.
		NCT04685135	3	Recruiting	KRYSTAL-12. Randomized, open-label study comparing monotherapy with docetaxel (taxane) in patients with advanced/metastatic NSCLC with KRAS^G12C^ mutation.
		NCT04975256	1/1b	Recruiting	KRYSTAL 14. Non-randomized, open-label study assessing MRTX849 in combination with BI 1701963 (SOS1 pan-KRAS inhibitor) in patients with advanced/metastatic solid malignancies (NSCLC or CRC) with KRAS^G12C^ mutation.
LY3499446	Eli Lilly and Company	NCT04165031	1/2	Terminated	Randomized, open label study comparing LY3499446 alone and in combination with abemaciclib, cetuximab, or erlotinib vs. docetaxel in patients with advanced/metastatic solid tumors with KRAS^G12C^ mutation. Study was terminated due to unexpected toxicity finding.
LY3537982	Eli Lilly and Company	NCT04956640	1	Not yet recruiting	Non-randomized, open-label study investigating LY3537982 alone and in combination with abemaciclib, erlotinib, sintilimab, temuterkib, LY3295668 (aurora A kinase inhibitor), or cetuximab in patients with KRAS^G12C^ mutant solid tumors.
GDC-6036	Genentech, Inc.	NCT04449874	1	Recruiting	Non-randomized, open-label study investigating GDC-6036 alone and in combination with atezolizumab, cetuximab, bevacizumab, erlotinib, or GDC-1971 (SHP2 inhibitor) in patients with advanced/metastatic solid tumors with KRAS^G12C^ mutation.
D-1553	InventisBio Inc.	NCT04585035	1/2	Recruiting	Non-randomized, open-label study evaluating D-1553 alone and in combination with other drugs used in standard treatment of solid tumors (not specified) in patients with advanced/metastatic solid tumors, including NSCLC and CRC, with KRAS^G12C^ mutation.
JDQ443	Novartis Pharmaceuticals	NCT04699188	1b/2	Recruiting	Non-randomized, open-label study assessing monotherapy and in combination with TNO155 (SHP2 inhibitor) and/or spartalizumab (anti-PD1 antibody) in patients with advanced (metastatic or unresectable) KRAS^G12C^ mutant solid tumors.
BI 1823911	Boehringer Ingelheim	NCT04973163	1a/1b	Not yet recruiting	Non-randomized, open-label study investigating BI 1823911 alone and in combination with BI 1701963 (SOS1::KRAS pan-KRAS inhibitor) in patients with advanced/metastatic solid tumors (e.g., NSCLC, CRC, pancreatic cancer, or cholangiocarcinoma) with KRAS^G12C^ mutation.

Abbreviations: *NSCLC* non-small cell lung cancer, *CRC* colorectal cancer

### AMG510

#### CodeBreaK100 (phase 1)

In August 2018, sotorasib (AMG510) became the first KRAS^G12C^ inhibitor both to enter human clinical trials and to demonstrate clinical safety and efficacy (NCT03600883, Table 1) [107, 108]. Hong and colleagues reported findings of a phase 1, multicenter, open-label trial of sotorasib in patients with heavily pretreated advanced (locally advanced or metastatic) solid tumors harboring the *KRAS* p.G12C mutation [107]. A total of 129 patients (59 NSCLC patients; 42 colorectal cancer [CRC] patients; and 28 patients with other tumor types) were enrolled in the dose escalation and expansion cohorts. Enrolled patients had received a median of 3 (range, 0 to 11) previous lines of anticancer therapies for metastatic disease. Sotorasib was administered orally once daily with each treatment cycle lasting 21 days. Because of its highest response, the 960 mg dosage administered daily was identified as the dose for the expansion cohort.

The primary endpoint was safety that included incidence of dose-limiting toxic effects (defined as sotorasib-related toxic effects within the first 21 days after the first dose), adverse events during the treatment period, and treatment-related adverse events. No dose-limiting toxic effects nor treatment-related adverse events resulting in death were observed. A total of 73 patients (56.6%) had treatment-related adverse events, and 15 patients (11.6%) had grade 3 or 4 events. The most common grade 3 treatment-related adverse events were gastrointestinal or hepatic side effects. Adverse events of any cause that occurred during treatment were reported in 125 patients (96.9%), with the most common events being diarrhea (29.5%), fatigue (23.3%), and nausea (20.9%). Grade 3 or higher adverse events of any cause that occurred during treatment were reported in 68 patients (52.7%).

Clinical efficacy was investigated as a secondary end point. Efficacy was described as objective response (complete or partial response), disease control (objective response or stable disease), duration of objective response, duration of stable disease, and progression-free survival. Among NSCLC patients, 19 patients (32.2%) had a confirmed objective response, and 52 patients (88.1%) had disease control. The median progression-free survival was 6.3 months. Among CRC patients, 3 patients (7.1%) had a confirmed response, and 31 patients (73.8%) had disease control. The median progression-free survival was 4.0 months.

Overall, the authors concluded that sotorasib had a relatively tolerable safety profile that consisted mainly of low-grade toxic effects and no dose-limiting toxic effects. Furthermore, sotorasib showed promising anticancer activity in patients with heavily pre-treated *KRAS* p.G12C mutant solid tumors, particularly NSCLC and CRC to a lesser extent.

#### CodeBreaK100 (phase 2)

In June 2021, Skoulidis et al. reported the results of the multicenter, single-group, open-label, phase 2 trial of CodeBreaK100 (NCT03600883, Table 1) [108]. The phase 2 trial evaluated the efficacy and safety of sotorasib monotherapy in patient with locally advanced or metastatic KRAS p.G12C-mutated NSCLC previously treated with standard therapies for *KRAS*-mutated NSCLC [108]. Among the 126 enrolled patients, the majority (81.0%) had previously received both platinum-based chemotherapy and inhibitors of PD-1 or PD-L1. Ultimately, 124 patients had measurable disease at baseline and were thus evaluated for response.

As the primary endpoint, an objective response was observed in 46 patients (37.1%; 95% confidence interval [CI]: 28.6 to 46.2), which exceeded their benchmark response of 23% in results previously reported in the REVEL trial investigating ramucirumab plus docetaxel in this population of patients [109]. Among the 46 patients, 4 (3.2%) had a complete response and 42 (33.9%) had a partial response. Examining secondary endpoints, disease control occurred in 100 patients (80.6%; 95% CI: 72.6 to 87.2). Additionally, among the 46 patients with an objective response, the median duration of response was 11.1 months (95% CI, 6.9 to could not be evaluated). Among the 124 patients, the median progression-free survival was 6.8 months (95% CI, 5.1 to 8.2), and the median overall survival of the entire 126 patient cohort was 12.5 months (95% CI, 10.0 to could not be evaluated).

Consistent with the safety findings of the phase 1 study, treatment with sotorasib in the phase 2 trial produced mostly grade 1 and 2 side effects, primarily low-grade hepatic and gastrointestinal toxic effects. Treatment-related adverse events occurred in 88 of 126 patients (69.8%), including grade 3 events in 25 patients (19.8%) and a grade 4 event in 1 (0.8%). No new safety signals were reported.

Exploratory biomarker analyses were conducted to assess associations between response to sotorasib therapy, baseline tumor PD-L1 expression level, tumor mutational burden, and mutations in *STK11*, *KEAP1*, and *TP53*, which are among the most prevalent genes with co-occurring mutations in *KRAS*-mutated NSCLC [110]. Of note, among all other subgroups or all patients that could be evaluated, the subgroup with *STK11*-mutated tumors and wild-type *KEAP1* represented the highest number of patients that responded to sotorasib. The current standard care for patients with newly diagnosed *KRAS*-mutated NSCLC commonly involves an immune-checkpoint inhibitor (e.g., PD-1/PD-L1 inhibitor) as monotherapy or in combination with chemotherapy [111–113]. Thus, this finding has important clinical implications since inactivating genomic alterations in *STK11* result in primary resistance to PD-1 and PD-L1 blockade and docetaxel in patients with *KRAS*-mutated NSCLC [114, 115].

Subsequent clinical trials are currently investigating sotorasib as monotherapy or in combination with various anticancer agents in patients with advanced or metastatic solid tumors, including NSCLC and CRC, with the KRAS p.G12C mutation (Table 1).

### MRTX849 (adagrasib)

In January 2019, adagrasib (MRTX849) entered the KRYSTAL-1 clinical trial, a multi-cohort phase 1/2 study in patients with advanced or metastatic solid tumors harboring a KRAS G12C mutation previously treated with chemotherapy and anti-PD-1/PD-L1 therapy (NCT03785249, Table 1). At the 32nd EORTC-NCI-AACR Symposium in October 2020, Jänne and colleagues reported the preliminary safety data for 79 patients with pretreated NSCLC who were treated with adagrasib 600 mg BID (i.e., twice daily) [116]. The most commonly reported (> 20%) treatment-related adverse events were nausea (54%), diarrhea (48%), vomiting (34%), fatigue (28%), and increased ALT (23%). The only commonly reported (> 2%) grade 3/4 treatment-related serious adverse event was hyponatremia (3%). In addition, among the 51 patients evaluable for clinical activity, 23 patients (45%) had an objective response, and 49 patients (96%) had disease control (i.e., 26/51 patients had stable disease). The median time on treatment was 8.2 months (range:1.4, 13.1+).

At the same conference, Johnson and colleagues reported KRYSTAL-1 preliminary safety data on 31 patients with CRC treated with adagrasib 600 mg BID [117]. The most commonly reported (> 20%) treatment-related adverse events include diarrhea (58%), nausea (52%), fatigue (42%), and vomiting (36%). Among the 18 CRC patients evaluable for clinical activity, 3 patients (17%) had a confirmed objective response, while 17 patients (94%) achieved disease control. Moreover, in the other solid tumor cohort with 6 evaluable patients for clinical activity, confirmed partial responses were observed in a patient with endometrial cancer and in another patient with pancreatic cancer. Unconfirmed partial responses were observed in two other patients with ovarian cancer and cholangiocarcinoma, respectively.

More recently, at the European Lung Cancer Virtual Congress 2021, Riely et al. presented updates on the NSCLC patients in the KRYSTAL-1 trial [118]. In addition to the data presented previously by Jänne et al., Riely et al. noted promising results in patients with STK-11 co-mutations who typically have a relatively poor response to immune checkpoint inhibition, as discussed earlier. Among these patients, 9/14 patients (64%) had an objective response. Examining the mechanistic action of adagrasib, preliminary pharmacodynamic and mechanistic biomarker analyses on pre- and post-treatment tumor NSCLC biopsies of 3 patients demonstrated downregulation of KRAS/MAPK pathway genes including *DUSP6* and *SPRY4*. Furthermore, in patients with tumors containing STK11-comutations, there was minimal expression of immune transcripts such as CD4 and CD8 at baseline. However, these immune transcripts were increased with adagrasib treatment, which suggests a potential immune response after therapy.

Subsequent clinical trials are currently investigating adagrasib as monotherapy or in combination with other anticancer agents in patients with advanced or metastatic solid tumors, including NSCLC and CRC, with the KRAS p.G12C mutation (Table 1).

### Other direct KRAS^G12C^ inhibitors

Additional clinical trials investigating other direct KRAS^G12C^ inhibitors are currently ongoing and are summarized below and in Table 1.

#### LY3499446, LY3537982

After the phase 1 clinical trial of LY3499446 was terminated due to an unexpected toxicity finding (NCT04165031), Eli Lilly and Company revealed LY3537982 at the AACR Annual Meeting 2021 [119]. According to Eli Lilly, LY3537982 is a potent, highly selective covalent inhibitor of the KRAS^G12C^ protein that was discovered using structure-based design. In preclinical studies, LY3537982 inhibited KRAS-GTP loading in the KRAS^G12C^ mutant H358 NSCLC cell line with an IC_50_ value of 3.35 nM, while AMG510 and MRTX849 had IC_50_ values of 47.9 nM and 89.9 nM, respectively. Moreover, LY3537982 selectively inhibited the growth of KRAS^G12C^ mutant tumor cells but not KRAS wild-type or non-G12C mutant cells. In multiple xenograft or patient-derived xenograft models with the KRAS^G12C^ mutation, LY3537982 had a range of anti-tumor activity ranging from complete regression to significant tumor growth inhibition. Furthermore, mechanism-based combinatory screens identified potential synergistic targeted therapies with LY3537982 that promoted enhanced anti-tumor activity in vitro and in vivo, including abemaciclib, LY3295668 (selective AurA inhibitor), and cetuximab. A phase 1 clinical trial evaluating the candidate LY3537982 is currently registered but not yet recruiting (NCT04956640). The trial is investigating LY3537982 alone and in combination with various therapies, including the aforementioned studied agents, in patients with KRAS^G12C^ mutant solid tumors.

#### GDC-6036

Created by Genentech, GDC-6036 is being investigated in a phase 1 clinical trial as monotherapy and in combination with other anti-cancer therapies in patients with advanced/metastatic solid tumors, including NSCLC and CRC, with a KRAS^G12C^ mutation (NCT04449874).

#### D-1553

Designed by InvestisBio, D-1553 was presented at the AACR Annual Meeting 2021 [120, 121]. According to the authors, D-1553 is an orally bioavailable, covalent small molecule compound that specifically inhibits KRAS^G12C^ in vitro (e.g., lung, pancreatic, colorectal cancer cell lines) and in vivo (e.g., cell line-derived and patient-derived xenograft tumor models). In both cell line-derived (CDX) and patient-derived xenograft (PDX) tumor models with KRAS^G12C^ mutation, D-1553 expressed anti-cancer activity as monotherapy and enhanced tumor growth inhibition or regression in combination with other targeted therapies (e.g., MEK inhibitor, SHP2 inhibitor) or chemotherapy. Specifically, in lung cancer PDX models, D-1553 exhibited tumor growth inhibition (TGI) ranging from 43.6 to 124.3%, with 4/8 models resulting in tumor regression. In colorectal cancer PDX models, TGI ranged from 60.9 to 105.7%, with 3/9 models showing tumor regression. An ongoing phase 1/2 clinical trial is evaluating D-1553 alone and in combination with other standard agents (not specified) in patients with advanced/metastatic solid tumors, including NSCLC and CRC, with the KRAS^G12C^ mutation (NCT04585035).

#### JDQ443

Created by Novartis Pharmaceuticals, JDQ443 is being examined in a phase 1/2 clinical trial as monotherapy and in combination with TNO155 (SHP2 inhibitor) and/or spartalizumab (anti-PD1 antibody) in patients with advanced or metastatic solid tumors with the KRAS^G12C^ mutation (NCT04699188).

#### BI 1823911

Designed by Boehringer Ingelheim, BI 1823911 is the latest small molecule KRAS^G12C^ inhibitor to enter clinical trials in July 2021 (NCT04973163). Initially presented at the AACR Annual Meeting 2021, BI 1823911 was found to have selective and potent anti-proliferative activity in a panel of KRAS^G12C^ mutant cancer cell lines with higher or similar potency compared to AMG510 and MRTX849 [64]. In a panel of KRAS^G12C^ NSCLC cell lines, treatment with BI 1823911 resulted in downregulation of MAPK pathway-responsive genes, such as *DUSP6* and *CCND1*. In addition, at the protein level, strong and sustained inactivation of the MAPK pathway was observed using p-ERK as a pharmacodynamic biomarker. Furthermore, in a panel of NSCLC or CRC CDX or PDX mouse models, a daily oral dose of 60 mg/kg BI 1823911 showed in vivo efficacy comparable to 100 mg/kg AMG510 and 100 mg/kg MRTX849, respectively. Finally, to examine potential synergistic anti-proliferative activity, the authors tested a large set of compounds in combination with BI 1823911 in a panel of selected KRAS^G12C^ mutant cancer cell lines. Among other MAPK and PI3K pathway inhibitors, the SOS1::KRAS inhibitor BI 1701963 was shown by the authors to be a promising combinatorial partner. BI 1701963 is a first-in-class, small molecule, pan-KRAS inhibitor that binds to and prevents SOS1 from associating with KRAS. Together, in vitro and in vivo combination studies with BI 1823911 and BI 1701963 in NSCLC and CRC tumor models showed increased, sustained pharmacodynamic modulation and stronger anti-tumor activity. A phase 1 clinical trial is underway investigating BI 1823911 alone and in combination with BI 1701963 in patients with advanced/metastatic solid tumors (e.g., NSCLC, CRC, pancreatic cancer, cholangiocarcinoma), with the KRAS^G12C^ mutation.

## Clinical mechanisms of adaptive resistance to direct KRAS^G12C^ inhibitors

Although mechanisms of resistance to direct KRAS^G12C^ inhibition have been explored in depth using preclinical models [96, 122–126], the clinical mechanisms of resistance remain to be elucidated. Awad et al. have begun to characterize the clinical mechanisms of resistance to adagrasib with results from the KRYSTAL-1 trial (NCT03785249) [95]. In the study, the authors included patients with KRAS^G12C^ mutant cancers who had disease progression while receiving adagrasib monotherapy. They defined acquired resistance to therapy as stable disease for at least 12 weeks or a partial or complete response followed by disease progression according to the Response Evaluation Criteria in Solid Tumors (RECIST), version 1.1. A total of 38 patients (27 NSCLC, 10 CRC, and 1 appendiceal cancer patients) were subsequently included in the study. Ultimately, putative mechanisms of resistance to adagrasib were detected in 17 patients (45%), of which 7 patients had multiple coincident mechanisms.

The authors categorized these putative resistance mechanisms into three main categories. First, secondary mutations or amplifications in *KRAS* were observed in patients. Specifically, acquired *KRAS* mutations were most notably found at codons 12, 13, 61, 68, 95, and 96; which included G12D/R/V/W, G13D, Q61H, R68S, H95D/Q/R, and Y96C. Codon 12 mutations prevented adagrasib binding, while the other secondary mutations occurred in the switch II drug-binding pocket. Secondly, another resistance mechanism is alternative oncogenic alterations that activate the RTK–RAS signaling pathway but do not directly alter KRAS itself. These acquired bypass mechanisms of resistance included *MET* amplification; activating mutations in *NRAS*, *BRAF*, *MAP 2 K1*, and *RET*; oncogenic fusions involving *ALK*, *RET*, *BRAF*, *RAF1*, and *FGFR3*; and loss-of-function mutations in *NF1* and *PTEN*. Thirdly, histologic transformation from lung adenocarcinoma to squamous-cell carcinoma was observed without identification of any other resistance mechanisms. Collectively, these data suggest that adagrasib treatments resulted in the evolution of a diverse set of adaptive mechanisms in KRAS^G12C^ mutant cancers, instead of dominant adaptive mechanisms seen with other targeted therapies.

## Conclusions

Thanks to the efforts of the Shokat lab in discovering the KRAS^G12C^ switch II pocket in 2013, it appears that we are currently in a golden era of developing targeted therapy that can directly inhibit the KRAS^G12C^-driven human cancers. As of early August 2021, twenty registered clinical trials have been implemented to investigate nine different direct KRAS^G12C^ inhibitors, which began with the first-in-human trial with sotorasib three years ago (Table 1). In addition to sotorasib (Lumakras™) becoming the first FDA-approved drug to directly target tumors with any *KRAS* mutation in May 2021, adagrasib received breakthrough therapy designation from the FDA in June 2021, thus making adagrasib one step closer to receiving FDA approval as well.

However, as with other clinical trials involving targeted therapies, such as the MEK inhibitors in *KRAS*-mutant NSCLC patients [127–133] and the BRAF inhibitors in *BRAF*^*V600E*^ mutant colorectal cancer patients [134–136], adaptive resistance to single-agent therapy with adagrasib and sotorasib eventually occurred in most patients. In the CodeBreaK100 phase 2 clinical trial with sotorasib, among the 126 patients with previously treated *KRAS* p.G12C-mutated NSCLC, 83 patients (65.9%) had discontinued treatment due to disease progression [108]. Analyzing results from the KRYSTAL-1 trial with adagrasib, Awad et al. initially characterized the clinical mechanisms of resistance to the direct KRAS^G12C^ inhibitors [95]. Their study supports the advent of novel KRAS inhibitors with different modes of binding and allelic specificities to counter the diversity of on-target and off-target adaptive resistance mechanisms. Moreover, effective combination therapy appears to be necessary to overcome these adaptive mechanisms of resistance to direct KRAS^G12C^ inhibitors. Clinical trials investigating various combination therapy modalities are currently undergoing (Table 1).

With the rapid and promising research and development of direct KRAS inhibitors, particularly against the KRAS^G12C^ mutant, there is further hope on the horizon for treating patients with cancers containing KRAS mutations.

## Data Availability

The dataset generated and/or analyzed during the current study is available in the Catalogue of Somatic Mutations in Cancer (COSMIC) repository, cancer.sanger.ac.uk. 10.1093/nar/gky1015

## Abbreviations

FDA: United States Food and Drug Administration
NSCLC: Non-small cell lung cancer
HVR: Hypervariable region
P-loop: Phosphate-binding loop
GDP: Guanosine diphosphate
GTP: Guanosine triphosphate
FTI: Farnesyltransferase inhibitor
RCE1: RAS-converting enzyme 1
ICMT: Isoprenylcysteine carboxymethyltransferase
GEF: Guanine nucleotide exchange factor
GAP: GTPase-activating protein
SOS: Son of sevenless homologue (SOS)
RASGRP: RAS guanyl nucelotide-releasing protein
COSMIC: Catalogue of Somatic Mutations in Cancer
TCGA: The Cancer Genome Atlas
MSKCC: Memorial Sloan Kettering Cancer Center
ICGC: International Cancer Genome Consortium
ACS: American Cancer Society
GENIE: Project Genomics Evidence Neoplasia Information Exchange
WT: Wild-type
RBD: Ras-binding domain
EGFR: Epidermal growth factor receptor
NCCN: National Comprehensive Cancer Network
S-IIP: Switch II pocket
RTK: Receptor tyrosine kinase
H95: Histidine 95
PD-1: Programmed cell death protein 1
PD-L1: Programmed death-ligand 1
CRC: Colorectal cancer
CI: Confidence interval
BID: Twice daily
CDX: Cell line-derived
PDX: Patient-derived xenograft
RECIST: Response Evaluation Criteria in Solid Tumors

## References

[1] Kirsten WH, Mayer LA (1967). Morphologic responses to a murine erythroblastosis virus. J Natl Cancer Inst.

[2] Scolnick EM, Rands E, Williams D, Parks WP (1973). Studies on the nucleic acid sequences of Kirsten sarcoma virus: a model for formation of a mammalian RNA-containing sarcoma virus. J Virol.

[3] Tsuchida N, Uesugi S (1981). Structure and functions of the Kirsten murine sarcoma virus genome: molecular cloning of biologically active Kirsten murine sarcoma virus DNA. J Virol.

[4] Ellis RW, Defeo D, Shih TY, Gonda MA, Young HA, Tsuchida N (1981). The p21 src genes of Harvey and Kirsten sarcoma viruses originate from divergent members of a family of normal vertebrate genes. Nature.

[5] Shih C, Shilo BZ, Goldfarb MP, Dannenberg A, Weinberg RA (1979). Passage of phenotypes of chemically transformed cells via transfection of DNA and chromatin. Proc Natl Acad Sci U S A.

[6] Shih C, Padhy LC, Murray M, Weinberg RA (1981). Transforming genes of carcinomas and neuroblastomas introduced into mouse fibroblasts. Nature.

[7] Perucho M, Goldfarb M, Shimizu K, Lama C, Fogh J, Wigler M (1981). Human-tumor-derived cell lines contain common and different transforming genes. Cell.

[8] Krontiris TG, Cooper GM (1981). Transforming activity of human tumor DNAs. Proc Natl Acad Sci U S A.

[9] Murray MJ, Shilo BZ, Shih C, Cowing D, Hsu HW, Weinberg RA (1981). Three different human tumor cell lines contain different oncogenes. Cell.

[10] Marshall CJ, Hall A, Weiss RA (1982). A transforming gene present in human sarcoma cell lines. Nature.

[11] Parada LF, Tabin CJ, Shih C, Weinberg RA (1982). Human EJ bladder carcinoma oncogene is homologue of Harvey sarcoma virus ras gene. Nature.

[12] Der CJ, Krontiris TG, Cooper GM (1982). Transforming genes of human bladder and lung carcinoma cell lines are homologous to the ras genes of Harvey and Kirsten sarcoma viruses. Proc Natl Acad Sci U S A.

[13] Santos E, Tronick SR, Aaronson SA, Pulciani S, Barbacid M (1982). T24 human bladder carcinoma oncogene is an activated form of the normal human homologue of BALB- and Harvey-MSV transforming genes. Nature.

[14] Parada LF, Weinberg RA (1983). Presence of a Kirsten murine sarcoma virus ras oncogene in cells transformed by 3-methylcholanthrene. Mol Cell Biol.

[15] Tabin CJ, Bradley SM, Bargmann CI, Weinberg RA, Papageorge AG, Scolnick EM (1982). Mechanism of activation of a human oncogene. Nature.

[16] Reddy EP, Reynolds RK, Santos E, Barbacid M (1982). A point mutation is responsible for the acquisition of transforming properties by the T24 human bladder carcinoma oncogene. Nature.

[17] Taparowsky E, Suard Y, Fasano O, Shimizu K, Goldfarb M, Wigler M (1982). Activation of the T24 bladder carcinoma transforming gene is linked to a single amino acid change. Nature.

[18] Capon DJ, Seeburg PH, McGrath JP, Hayflick JS, Edman U, Levinson AD (1983). Activation of Ki-ras2 gene in human colon and lung carcinomas by two different point mutations. Nature.

[19] Cox AD, Der CJ (2010). Ras history: the saga continues. Small GTPases.

[20] Santos E, Martin-Zanca D, Reddy EP, Pierotti MA, Della Porta G, Barbacid M (1984). Malignant activation of a K-ras oncogene in lung carcinoma but not in normal tissue of the same patient. Science.

[21] Feig LA, Bast RC, Knapp RC, Cooper GM (1984). Somatic activation of rasK gene in a human ovarian carcinoma. Science.

[22] Malumbres M, Barbacid M (2003). RAS oncogenes: the first 30 years. Nat Rev Cancer.

[23] Tsuchida N, Murugan AK, Grieco M (2016). Kirsten Ras* oncogene: significance of its discovery in human cancer research. Oncotarget.

[24] Ostrem JM, Shokat KM (2016). Direct small-molecule inhibitors of KRAS: from structural insights to mechanism-based design. Nat Rev Drug Discov.

[25] Lampson BL, Pershing NL, Prinz JA, Lacsina JR, Marzluff WF, Nicchitta CV (2013). Rare codons regulate KRas oncogenesis. Curr Biol.

[26] Vetter IR, Wittinghofer A (2001). The guanine nucleotide-binding switch in three dimensions. Science.

[27] Abankwa D, Gorfe AA, Inder K, Hancock JF (2010). Ras membrane orientation and nanodomain localization generate isoform diversity. Proc Natl Acad Sci U S A.

[28] Simanshu DK, Nissley DV, McCormick F (2017). RAS proteins and their regulators in human disease. Cell.

[29] Lobell RB, Liu D, Buser CA, Davide JP, DePuy E, Hamilton K (2002). Preclinical and clinical pharmacodynamic assessment of L-778,123, a dual inhibitor of farnesyl:protein transferase and geranylgeranyl:protein transferase type-I. Mol Cancer Ther.

[30] Uprety D, Adjei AA (2020). KRAS: from undruggable to a druggable cancer target. Cancer Treat Rev.

[31] Liu P, Wang Y, Li X (2019). Targeting the untargetable KRAS in cancer therapy. Acta Pharm Sin B.

[32] Downward J (2003). Targeting RAS signalling pathways in cancer therapy. Nat Rev Cancer.

[33] Jancik S, Drabek J, Radzioch D, Hajduch M (2010). Clinical relevance of KRAS in human cancers. J Biomed Biotechnol.

[34] Ryan MB, Corcoran RB (2018). Therapeutic strategies to target RAS-mutant cancers. Nat Rev Clin Oncol.

[35] Mattox TE, Chen X, Maxuitenko YY, Keeton AB, Piazza GA (2019). Exploiting RAS nucleotide cycling as a strategy for drugging RAS-driven cancers. Int J Mol Sci.

[36] Hunter JC, Manandhar A, Carrasco MA, Gurbani D, Gondi S, Westover KD (2015). Biochemical and structural analysis of common Cancer-associated KRAS mutations. Mol Cancer Res.

[37] Boriack-Sjodin PA, Margarit SM, Bar-Sagi D, Kuriyan J (1998). The structural basis of the activation of Ras by Sos. Nature.

[38] Hobbs GA, Der CJ, Rossman KL (2016). RAS isoforms and mutations in cancer at a glance. J Cell Sci.

[39] Tate JG, Bamford S, Jubb HC, Sondka Z, Beare DM, Bindal N (2019). COSMIC: the catalogue of somatic mutations in Cancer. Nucleic Acids Res.

[40] Sanchez-Vega F, Mina M, Armenia J, Chatila WK, Luna A, La KC (2018). Oncogenic signaling pathways in the Cancer genome atlas. Cell.

[41] Cerami E, Gao J, Dogrusoz U, Gross BE, Sumer SO, Aksoy BA (2012). The cBio cancer genomics portal: an open platform for exploring multidimensional cancer genomics data. Cancer Discov.

[42] Zhang J, Bajari R, Andric D, Gerthoffert F, Lepsa A, Nahal-Bose H (2019). The International Cancer Genome Consortium data portal. Nat Biotechnol.

[43] Prior IA, Hood FE, Hartley JL (2020). The frequency of Ras mutations in cancer. Cancer Res.

[44] Bray F, Ferlay J, Soerjomataram I, Siegel RL, Torre LA, Jemal A (2018). Global cancer statistics 2018: GLOBOCAN estimates of incidence and mortality worldwide for 36 cancers in 185 countries. CA Cancer J Clin.

[45] Prior IA, Lewis PD, Mattos C (2012). A comprehensive survey of Ras mutations in cancer. Cancer Res.

[46] Gao J, Aksoy BA, Dogrusoz U, Dresdner G, Gross B, Sumer SO (2013). Integrative analysis of complex cancer genomics and clinical profiles using the cBioPortal. Sci Signal.

[47] Cox AD, Fesik SW, Kimmelman AC, Luo J, Der CJ (2014). Drugging the undruggable RAS: mission possible?. Nat Rev Drug Discov.

[48] Nassar AH, Adib E, Kwiatkowski DJ (2021). Distribution of KRAS (G12C) somatic mutations across race, sex, and Cancer type. N Engl J Med.

[49] Consortium APG (2017). AACR project GENIE: powering precision medicine through an international consortium. Cancer Discov.

[50] Scheffzek K, Ahmadian MR, Kabsch W, Wiesmuller L, Lautwein A, Schmitz F (1997). The Ras-RasGAP complex: structural basis for GTPase activation and its loss in oncogenic Ras mutants. Science.

[51] Seeburg PH, Colby WW, Capon DJ, Goeddel DV, Levinson AD (1984). Biological properties of human c-ha-ras1 genes mutated at codon 12. Nature.

[52] Der CJ, Finkel T, Cooper GM (1986). Biological and biochemical properties of human rasH genes mutated at codon 61. Cell.

[53] Smith MJ, Neel BG, Ikura M (2013). NMR-based functional profiling of RASopathies and oncogenic RAS mutations. Proc Natl Acad Sci U S A.

[54] Ihle NT, Byers LA, Kim ES, Saintigny P, Lee JJ, Blumenschein GR (2012). Effect of KRAS oncogene substitutions on protein behavior: implications for signaling and clinical outcome. J Natl Cancer Inst.

[55] Hammond DE, Mageean CJ, Rusilowicz EV, Wickenden JA, Clague MJ, Prior IA (2015). Differential reprogramming of isogenic colorectal cancer cells by distinct activating KRAS mutations. J Proteome Res.

[56] Lievre A, Bachet JB, Le Corre D, Boige V, Landi B, Emile JF (2006). KRAS mutation status is predictive of response to cetuximab therapy in colorectal cancer. Cancer Res.

[57] Linardou H, Dahabreh IJ, Kanaloupiti D, Siannis F, Bafaloukos D, Kosmidis P (2008). Assessment of somatic k-RAS mutations as a mechanism associated with resistance to EGFR-targeted agents: a systematic review and meta-analysis of studies in advanced non-small-cell lung cancer and metastatic colorectal cancer. Lancet Oncol.

[58] Douillard JY, Oliner KS, Siena S, Tabernero J, Burkes R, Barugel M (2013). Panitumumab-FOLFOX4 treatment and RAS mutations in colorectal cancer. N Engl J Med.

[59] Karapetis CS, Khambata-Ford S, Jonker DJ, O'Callaghan CJ, Tu D, Tebbutt NC (2008). K-ras mutations and benefit from cetuximab in advanced colorectal cancer. N Engl J Med.

[60] Peeters M, Oliner KS, Price TJ, Cervantes A, Sobrero AF, Ducreux M (2015). Analysis of KRAS/NRAS mutations in a phase III study of panitumumab with FOLFIRI compared with FOLFIRI alone as second-line treatment for metastatic colorectal cancer. Clin Cancer Res.

[61] Li QH, Wang YZ, Tu J, Liu CW, Yuan YJ, Lin R (2020). Anti-EGFR therapy in metastatic colorectal cancer: mechanisms and potential regimens of drug resistance. Gastroenterol Rep (Oxf).

[62] Allegra CJ, Jessup JM, Somerfield MR, Hamilton SR, Hammond EH, Hayes DF (2009). American Society of Clinical Oncology provisional clinical opinion: testing for KRAS gene mutations in patients with metastatic colorectal carcinoma to predict response to anti-epidermal growth factor receptor monoclonal antibody therapy. J Clin Oncol.

[63] Tran NH, Cavalcante LL, Lubner SJ, Mulkerin DL, LoConte NK, Clipson L (2015). Precision medicine in colorectal cancer: the molecular profile alters treatment strategies. Ther Adv Med Oncol.

[64] Savarese F, Gollner A, Rudolph D, Lipp J, Popow J, Hofmann MH, et al. In vitro and in vivo characterization of BI 1823911 - a novel KRASG12C selective small molecule inhibitor [abstract]. Cancer Res. 2021;81(13_Suppl) Abstract nr 1271. 10.1158/1538-7445.AM2019-2707.

[65] Keeton AB, Ward A, Chen X, Valiyaveettil J, Zhu B, Ramirez-Alcantara V, et al. A novel RAS inhibitor, MCI-062, inhibits colon tumor growth in vivo and activates antitumor immunity [abstract]. Cancer Res. 2019;79(13 Suppl) Abstract nr 2707. 10.1158/1538-7445.AM2021-1271.

[66] Nagasaka M, Li Y, Sukari A, Ou SI, Al-Hallak MN, Azmi AS (2020). KRAS G12C game of thrones, which direct KRAS inhibitor will claim the iron throne?. Cancer Treat Rev.

[67] Christensen JG, Olson P, Briere T, Wiel C, Bergo MO (2020). Targeting Kras(g12c) -mutant cancer with a mutation-specific inhibitor. J Intern Med.

[68] Papke B, Der CJ (2017). Drugging RAS: know the enemy. Science.

[69] Stalnecker CA, Der CJ. RAS, wanted dead or alive: advances in targeting RAS mutant cancers. Sci Signal. 2020;13(624). 10.1126/scisignal.aay6013. 10.1126/scisignal.aay6013 PMC7393681 32209699

[70] Blume-Jensen P, Hunter T (2001). Oncogenic kinase signalling. Nature.

[71] Spiegel J, Cromm PM, Zimmermann G, Grossmann TN, Waldmann H (2014). Small-molecule modulation of Ras signaling. Nat Chem Biol.

[72] Serna-Blasco R, Sanz-Alvarez M, Aguilera O, Garcia-Foncillas J (2019). Targeting the RAS-dependent chemoresistance: the Warburg connection. Semin Cancer Biol.

[73] Ni D, Li X, He X, Zhang H, Zhang J, Lu S (2019). Drugging K-Ras(G12C) through covalent inhibitors: Mission possible?. Pharmacol Ther.

[74] McCormick F (2019). Progress in targeting RAS with small molecule drugs. Biochem J.

[75] Waters AM, Der CJ (2018). KRAS: the critical driver and therapeutic target for pancreatic cancer. Cold Spring Harb Perspect Med.

[76] Downward J (2015). RAS synthetic lethal screens revisited: still seeking the elusive prize?. Clin Cancer Res.

[77] Kaelin WG (2005). The concept of synthetic lethality in the context of anticancer therapy. Nat Rev Cancer.

[78] McCormick F (2015). KRAS as a therapeutic target. Clin Cancer Res.

[79] Indini A, Rijavec E, Ghidini M, Cortellini A, Grossi F (2021). Targeting KRAS in solid tumors: current challenges and future opportunities of novel KRAS inhibitors. Pharmaceutics.

[80] Mai TT, Lito P (2018). A treatment strategy for KRAS-driven tumors. Nat Med.

[81] McCormick F (2018). Targeting KRAS directly. Ann Rev Cancer Biol.

[82] Patricelli MP, Janes MR, Li LS, Hansen R, Peters U, Kessler LV (2016). Selective inhibition of oncogenic KRAS output with small molecules targeting the inactive state. Cancer Discov.

[83] Lito P, Solomon M, Li LS, Hansen R, Rosen N (2016). Allele-specific inhibitors inactivate mutant KRAS G12C by a trapping mechanism. Science.

[84] Janes MR, Zhang J, Li LS, Hansen R, Peters U, Guo X (2018). Targeting KRAS mutant cancers with a covalent G12C-specific inhibitor. Cell.

[85] Lanman BA, Allen JR, Allen JG, Amegadzie AK, Ashton KS, Booker SK (2020). Discovery of a covalent inhibitor of KRAS(G12C) (AMG 510) for the treatment of solid tumors. J Med Chem.

[86] Shin Y, Jeong JW, Wurz RP, Achanta P, Arvedson T, Bartberger MD (2019). Discovery of N-(1-Acryloylazetidin-3-yl)-2-(1H-indol-1-yl) acetamides as covalent inhibitors of KRAS(G12C). ACS Med Chem Lett.

[87] Gentile DR, Rathinaswamy MK, Jenkins ML, Moss SM, Siempelkamp BD, Renslo AR (2017). Ras binder induces a modified switch-II pocket in GTP and GDP states.. Cell Chem Biol.

[88] Canon J, Rex K, Saiki AY, Mohr C, Cooke K, Bagal D (2019). The clinical KRAS(G12C) inhibitor AMG 510 drives anti-tumour immunity. Nature.

[89] Robert C, Karaszewska B, Schachter J, Rutkowski P, Mackiewicz A, Stroiakovski D (2015). Improved overall survival in melanoma with combined dabrafenib and trametinib. N Engl J Med.

[90] Ribas A, Wolchok JD (2018). Cancer immunotherapy using checkpoint blockade. Science.

[91] Marin-Acevedo JA, Kimbrough EO, Lou Y (2021). Next generation of immune checkpoint inhibitors and beyond. J Hematol Oncol.

[92] Spranger S, Dai D, Horton B, Gajewski TF (2017). Tumor-residing Batf3 dendritic cells are required for effector T cell trafficking and adoptive T cell therapy. Cancer Cell.

[93] Gao Q, Wang S, Chen X, Cheng S, Zhang Z, Li F (2019). Cancer-cell-secreted CXCL11 promoted CD8(+) T cells infiltration through docetaxel-induced-release of HMGB1 in NSCLC. J Immunother Cancer.

[94] Ebert PJR, Cheung J, Yang Y, McNamara E, Hong R, Moskalenko M (2016). MAP kinase inhibition promotes T cell and anti-tumor activity in combination with PD-L1 checkpoint blockade. Immunity.

[95] Awad MM, Liu S, Rybkin II, Arbour KC, Dilly J, Zhu VW (2021). Acquired resistance to KRAS(G12C) inhibition in cancer. N Engl J Med.

[96] Xue JY, Zhao Y, Aronowitz J, Mai TT, Vides A, Qeriqi B (2020). Rapid non-uniform adaptation to conformation-specific KRAS(G12C) inhibition. Nature.

[97] Scheffler M, Ihle MA, Hein R, Merkelbach-Bruse S, Scheel AH, Siemanowski J (2019). K-ras mutation subtypes in NSCLC and associated co-occuring mutations in other oncogenic pathways. J Thorac Oncol.

[98] Richman SD, Chambers P, Seymour MT, Daly C, Grant S, Hemmings G (2011). Intra-tumoral heterogeneity of KRAS and BRAF mutation status in patients with advanced colorectal cancer (aCRC) and cost-effectiveness of multiple sample testing. Anal Cell Pathol (Amst).

[99] Lamy A, Blanchard F, Le Pessot F, Sesboue R, Di Fiore F, Bossut J (2011). Metastatic colorectal cancer KRAS genotyping in routine practice: results and pitfalls. Mod Pathol.

[100] Kordiak J, Szemraj J, Grabska-Kobylecka I, Bialasiewicz P, Braun M, Kordek R (2019). Intratumor heterogeneity and tissue distribution of KRAS mutation in non-small cell lung cancer: implications for detection of mutated KRAS oncogene in exhaled breath condensate. J Cancer Res Clin Oncol.

[101] Fell JB, Fischer JP, Baer BR, Ballard J, Blake JF, Bouhana K (2018). Discovery of tetrahydropyridopyrimidines as irreversible covalent inhibitors of KRAS-G12C with in vivo activity. ACS Med Chem Lett.

[102] Gabizon R, London N (2020). Hitting KRAS when it's down. J Med Chem.

[103] Fell JB, Fischer JP, Baer BR, Blake JF, Bouhana K, Briere DM (2020). Identification of the clinical development candidate MRTX849, a covalent KRAS(G12C) inhibitor for the treatment of cancer. J Med Chem.

[104] Hallin J, Engstrom LD, Hargis L, Calinisan A, Aranda R, Briere DM (2020). The KRAS(G12C) inhibitor MRTX849 provides insight toward therapeutic susceptibility of KRAS-mutant cancers in mouse models and patients. Cancer Discov.

[105] Misale S, Fatherree JP, Cortez E, Li C, Bilton S, Timonina D (2019). KRAS G12C NSCLC models are sensitive to direct targeting of KRAS in combination with PI3K inhibition. Clin Cancer Res.

[106] Lou K, Steri V, Ge AY, Hwang YC, Yogodzinski CH, Shkedi AR (2019). KRAS(G12C) inhibition produces a driver-limited state revealing collateral dependencies. Sci Signal.

[107] Hong DS, Fakih MG, Strickler JH, Desai J, Durm GA, Shapiro GI (2020). KRAS(G12C) inhibition with sotorasib in advanced solid tumors. N Engl J Med.

[108] Skoulidis F, Li BT, Dy GK, Price TJ, Falchook GS, Wolf J (2021). Sotorasib for lung cancers with KRAS p.G12C mutation. N Engl J Med.

[109] Garon EB, Ciuleanu TE, Arrieta O, Prabhash K, Syrigos KN, Goksel T (2014). Ramucirumab plus docetaxel versus placebo plus docetaxel for second-line treatment of stage IV non-small-cell lung cancer after disease progression on platinum-based therapy (REVEL): a multicentre, double-blind, randomised phase 3 trial. Lancet.

[110] Arbour KC, Jordan E, Kim HR, Dienstag J, Yu HA, Sanchez-Vega F (2018). Effects of co-occurring genomic alterations on outcomes in patients with KRAS-mutant non-small cell lung cancer. Clin Cancer Res.

[111] Carbone DP, Reck M, Paz-Ares L, Creelan B, Horn L, Steins M (2017). First-line nivolumab in stage IV or recurrent non-small-cell lung cancer. N Engl J Med.

[112] Gandhi L, Rodriguez-Abreu D, Gadgeel S, Esteban E, Felip E, De Angelis F (2018). Pembrolizumab plus chemotherapy in metastatic non-small-cell lung cancer. N Engl J Med.

[113] Mok TSK, Wu YL, Kudaba I, Kowalski DM, Cho BC, Turna HZ (2019). Pembrolizumab versus chemotherapy for previously untreated, PD-L1-expressing, locally advanced or metastatic non-small-cell lung cancer (KEYNOTE-042): a randomised, open-label, controlled, phase 3 trial. Lancet.

[114] Singh A, Daemen A, Nickles D, Jeon SM, Foreman O, Sudini K (2021). NRF2 activation promotes aggressive lung cancer and associates with poor clinical outcomes. Clin Cancer Res.

[115] Skoulidis F, Goldberg ME, Greenawalt DM, Hellmann MD, Awad MM, Gainor JF (2018). STK11/LKB1 mutations and PD-1 inhibitor resistance in KRAS-mutant lung adenocarcinoma. Cancer Discov.

[116] Jänne PA, Rybkin II, Riely GJ, Papadopoulos KP, Sabari JK, Johnson ML (2020). 3LBA late breaking - KRYSTAL-1: activity and safety of adagrasib (MRTX849) in advanced/metastatic non–small-cell lung cancer (NSCLC) harboring KRAS G12C mutation. Eur J Cancer.

[117] Johnson ML, Ou SHI, Barve M, Rybkin II, Papadopoulos KP, Leal TA (2020). 4LBA late breaking - KRYSTAL-1: activity and safety of adagrasib (MRTX849) in patients with colorectal cancer (CRC) and other solid tumors harboring a KRAS G12C mutation. Eur J Cancer.

[118] Riely GJ, Ou S-HI, Rybkin I, Spira A, Papadopoulos K, Sabari JK (2021). 99O_PR KRYSTAL-1: activity and preliminary pharmacodynamic (PD) analysis of adagrasib (MRTX849) in patients (Pts) with advanced non–small cell lung cancer (NSCLC) harboring KRASG12C mutation. J Thorac Oncol.

[119] Peng S-B, Si C, Zhang Y, Horn RDV, Lin X, Gong X, et al. Preclinical characterization of LY3537982, a novel, highly selective and potent KRAS-G12C inhibitor [abstract]. Cancer Res. 2021;81(13_Suppl) Abstract nr 1259. 10.1158/1538-7445.AM2021-1259.

[120] Shi Z, Weng J, Fan X, Wang E, Zhu Q, Tao L, et al. Discovery of D-1553, a novel and selective KRas-G12C inhibitor with potent anti-tumor activity in a broad spectrum of tumor cell lines and xenograft models [abstract]. Cancer Res. 2021;81(13_Suppl) Abstract nr 932. 10.1158/1538-7445.AM2021-932.

[121] Shi Z, Weng J, Fan X, Zhu Q, Robb E, Moriarty A, et al. Potent in vivo anti-tumor activity of D-1553 as a single agent and in combination with targeted therapeutics in a broad spectrum of patient-derived xenograft tumor models with KRas G12C mutation [abstract]. Cancer Res. 2021;81(13_Suppl) Abstract nr 1056. 10.1158/1538-7445.AM2021-1056.

[122] Jiao D, Yang S. Overcoming resistance to drugs targeting KRAS(G12C) mutation. Innovation (N Y). 2020;1(2). 10.1016/j.xinn.2020.100035. 10.1016/j.xinn.2020.100035 PMC7491749 32939510

[123] Hata AN, Shaw AT (2020). Resistance looms for KRAS(G12C) inhibitors. Nat Med.

[124] Dunnett-Kane V, Nicola P, Blackhall F, Lindsay C. Mechanisms of resistance to KRAS(G12C) inhibitors. Cancers (Basel). 2021;13(1). 10.3390/cancers13010151. 10.3390/cancers13010151 PMC7795113 33466360

[125] Amodio V, Yaeger R, Arcella P, Cancelliere C, Lamba S, Lorenzato A (2020). EGFR blockade reverts resistance to KRAS(G12C) inhibition in colorectal cancer. Cancer Discov.

[126] Ryan MB, Fece de la Cruz F, Phat S, Myers DT, Wong E, Shahzade HA (2020). Vertical pathway inhibition overcomes adaptive feedback resistance to KRAS(G12C) inhibition. Clin Cancer Res.

[127] Kun E, Tsang YTM, Ng CW, Gershenson DM, Wong KK (2021). MEK inhibitor resistance mechanisms and recent developments in combination trials. Cancer Treat Rev.

[128] Caunt CJ, Sale MJ, Smith PD, Cook SJ (2015). MEK1 and MEK2 inhibitors and cancer therapy: the long and winding road. Nat Rev Cancer.

[129] Blumenschein GR, Smit EF, Planchard D, Kim DW, Cadranel J, De Pas T (2015). A randomized phase II study of the MEK1/MEK2 inhibitor trametinib (GSK1120212) compared with docetaxel in KRAS-mutant advanced non-small-cell lung cancer (NSCLC). Ann Oncol.

[130] Janne PA, van den Heuvel MM, Barlesi F, Cobo M, Mazieres J, Crino L (2017). Selumetinib plus docetaxel compared with docetaxel alone and progression-free survival in patients with KRAS-mutant advanced non-small cell lung cancer: the SELECT-1 randomized clinical trial. JAMA.

[131] Infante JR, Fecher LA, Falchook GS, Nallapareddy S, Gordon MS, Becerra C (2012). Safety, pharmacokinetic, pharmacodynamic, and efficacy data for the oral MEK inhibitor trametinib: a phase 1 dose-escalation trial. Lancet Oncol.

[132] Kitai H, Ebi H, Tomida S, Floros KV, Kotani H, Adachi Y (2016). Epithelial-to-mesenchymal transition defines feedback activation of receptor tyrosine kinase signaling induced by MEK inhibition in KRAS-mutant lung cancer. Cancer Discov.

[133] Ambrogio C, Kohler J, Zhou ZW, Wang H, Paranal R, Li J (2018). KRAS dimerization impacts MEK inhibitor sensitivity and oncogenic activity of mutant KRAS. Cell.

[134] Corcoran RB, Ebi H, Turke AB, Coffee EM, Nishino M, Cogdill AP (2012). EGFR-mediated re-activation of MAPK signaling contributes to insensitivity of BRAF mutant colorectal cancers to RAF inhibition with vemurafenib. Cancer Discov.

[135] Prahallad A, Sun C, Huang S, Di Nicolantonio F, Salazar R, Zecchin D (2012). Unresponsiveness of colon cancer to BRAF(V600E) inhibition through feedback activation of EGFR. Nature.

[136] Kopetz S, Desai J, Chan E, Hecht JR, O'Dwyer PJ, Lee RJ (2010). PLX4032 in metastatic colorectal cancer patients with mutant BRAF tumors. J Clin Oncol.

